# Leading-process actomyosin coordinates organelle positioning and adhesion receptor dynamics in radially migrating cerebellar granule neurons

**DOI:** 10.1186/1749-8104-9-26

**Published:** 2014-12-02

**Authors:** Niraj Trivedi, Joseph S Ramahi, Mahmut Karakaya, Danielle Howell, Ryan A Kerekes, David J Solecki

**Affiliations:** Department of Developmental Neurobiology, St. Jude Children’s Research Hospital, 262 Danny Thomas Place, Memphis, TN 38105 USA; Imaging, Signals and Machine Learning Group, Oak Ridge National Laboratory, Oak Ridge, TN 37831 USA

## Abstract

**Background:**

During brain development, neurons migrate from germinal zones to their final positions to assemble neural circuits. A unique saltatory cadence involving cyclical organelle movement (e.g., centrosome motility) and leading-process actomyosin enrichment prior to nucleokinesis organizes neuronal migration. While functional evidence suggests that leading-process actomyosin is essential for centrosome motility, the role of the actin-enriched leading process in globally organizing organelle transport or traction forces remains unexplored.

**Results:**

We show that myosin ii motors and F-actin dynamics are required for Golgi apparatus positioning before nucleokinesis in cerebellar granule neurons (CGNs) migrating along glial fibers. Moreover, we show that primary cilia are motile organelles, localized to the leading-process F-actin-rich domain and immobilized by pharmacological inhibition of myosin ii and F-actin dynamics. Finally, leading process adhesion dynamics are dependent on myosin ii and F-actin.

**Conclusions:**

We propose that actomyosin coordinates the overall polarity of migrating CGNs by controlling asymmetric organelle positioning and cell-cell contacts as these cells move along their glial guides.

**Electronic supplementary material:**

The online version of this article (doi:10.1186/1749-8104-9-26) contains supplementary material, which is available to authorized users.

## Background

The necessity of neuronal migration for appropriate nervous system lamination and circuit formation has spurred intense investigation into the molecular and cellular mechanisms of this crucial morphogenic movement [1–3]. In most brain regions, neurons use a stereotypical saltatory motility cycle involving a sequential organelle transport and adhesion/de-adhesion events to migrate along their substrates [4–10]. They first elaborate a leading process that adheres to substrates (e.g., glial cells, axons) and guides the direction of migration. Next, in some populations of migrating neurons, including cerebellar granule neurons (CGNs), pyramidal neurons, and gonadotropin-releasing hormone neurons, an F-actin- and myosin ii motor-enriched region of the leading process proximal to the neuronal soma [11–15] (sometimes called the cytoplasmic dilation [16]) becomes engorged with cytoplasmic components, including the centrosome and Golgi apparatus [17–24]. After the centrosome translocates through the leading process, the nucleus follows and the sequence is repeated until the neuron reaches its destined cortical lamina. The significance of this two-stroke sequence is illustrated by its conservation in neurons throughout the vertebrate brain and by its apparent requirement for appropriate migration, as perturbation of cytoskeletal and signaling components essential for migration strongly affect the choreography of the motility cycle [13, 15, 19, 21–23, 25].

The two-stroke nucleokinesis cycle has served as the main model for studies of the polarity of migrating neurons and the spatiotemporal roles of cytoskeletal components in migration. While disturbance of the microtubule and actin cytoskeletons is known to perturb the two-stroke cycle, only recently have time-lapse imaging studies provided mechanistic insight into the coordination of the motility cycle. Cytoplasmic dynein motors are localized at the base of the neuron’s leading process and soma, where they are thought to generate pulling forces on microtubules that help position the centrosome and facilitate subsequent somal translocation [22]. The leading process is also a site of F-actin dynamics accumulation and myosin ii motor activity [11–13, 15]. Myosin ii-powered actin flow in the direction of migration is required for centrosome positioning and eventual somal translocation.

Despite these advances, it has remained unexplored whether the cytoskeletal forces that position the centrosome are unique to this organelle or apply more broadly to other events linked to two-stroke motility cycle and ultimately to the polarity of migrating neurons. We were curious whether leading-process actomyosin cytoskeleton coordinates the positioning of other cytoplasmic organelles, how organelle positioning is coordinated with substrate adhesion, and whether actomyosin organizes the overall polarity of migrating neurons. We generated a panel of time-lapse imaging probes to examine, for the first time, the dynamic distribution of the Golgi apparatus, primary cilia, and cell-cell adhesions in cultured CGNs migrating along glial fibers – a well-established model for radial neuronal migration. We used time-lapse imaging to mechanistically test the hypothesis that leading-process actomyosin controls both global organelle positioning and the loci of adhesive traction in the leading process. We show that the motility of the Golgi apparatus, which has been postulated via examination of fixed neurons to undergo two-stroke movement, depends on myosin ii motor activity. Further, the polarized transport of the primary cilia (consistent with the two-stroke cycle) requires myosin ii motors and F-actin cytoskeletal dynamics. Finally, we found that the formation and turnover of adhesions in the F-actin-enriched region of the leading process and soma requires the actomyosin cytoskeleton. Imaging of adhesion dynamics revealed that the neuronal soma appears to slide past adhesions before their final disassembly in the CGN trailing process, suggesting adhesion disassembly is not precisely linked to somal advance. Taken together, these findings demonstrate that the leading-process actomyosin traction forces not only position multiple cytoplasmic organelles but also regulate adhesion formation and turnover that precedes somal translocation. Considering that asymmetric organelle transport and cell-cell adhesion distributions are hallmarks of cell polarity, we propose that myosin ii no longer be viewed only as a motor that powers cytoskeletal arrangement for neuronal migration; instead, we propose that it be viewed as broadly coordinating the overall polarity of migrating neurons.

## Results and discussion

### The Golgi apparatus displays two-stroke motility that parallels F-actin dynamics

**Figure 1:**
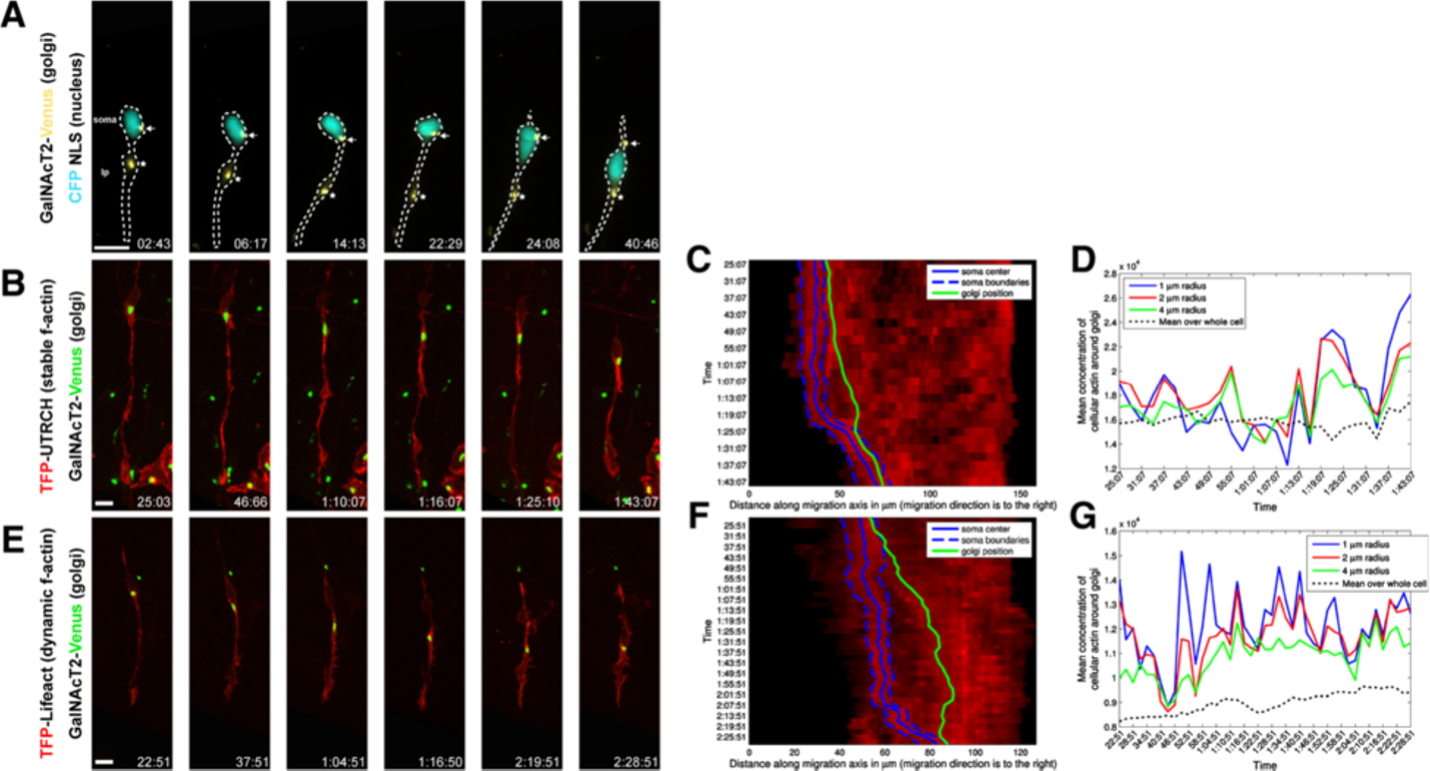
**The Golgi apparatus displays two-stroke motility dynamics.** **(A)** The Golgi apparatus (labeled by GalNAcT2-Venus, yellow) translocates before the nucleus (labeled by cyan fluorescent protein-NLS, teal) moves forward. The soma and leading process are labeled in the first frame and a star indicates the position of a Golgi fragment in the leading process dilation and an arrow indicates a Golgi fragment in the cell body. The Golgi apparatus (green) is embedded within the stable F-actin (labeled by **(B)** TFP-UTRCH, red, and by **(E)** TFP-Lifeact, red) contractile domain in the leading process of living neurons. Note that the Golgi enters the leading process when the process’ base dilates. **(C, F)** Adaptive volumetric kymographs of the sequences shown in Panels B and E (somal boundaries, dashed blue line; soma center, solid blue line; Golgi position, green line; TFP-UTRCH or TFP-Lifeact, red). **(D, G)** Analysis of mean actin concentration around the Golgi for the sequences shown in Panels B and E, concentrations were computed within a 1, 2, or 4 μm radius from the center of the Golgi (black dashed line represents the average actin concentration throughout the cell). Scale bar, 10 μm.

We used the two-stroke nucleokinesis model, which is the usual frame of reference for dissecting the cytoskeletal components required for positioning of the organelles and nucleus during migration. Despite intense study of the contribution of actin- and microtubule-based motors to the two-stroke cycle, particularly centrosome motility, it remains unclear whether additional organelle positioning events linked to the polarity of neuronal migration require actomyosin contractility. To address this question, we examined the positioning of the Golgi apparatus in CGNs migrating along Bergmann glial fibers *in vitro*, as the Golgi (like the centrosome) moves into the leading process before somal translocation in migrating medial ganglionic eminence (MGE) interneurons and cortical pyramidal neurons [20, 24]. Time-lapse imaging of CGNs expressing cyan fluorescent protein tagged with a nuclear localization signal and GalNAcT2-YFP (yellow fluorescent protein, a Golgi apparatus probe) [26], revealed that the Golgi behaves similarly to the centrosome, entering a dilated region near the proximal portion of the leading process before nuclear translocation (Figure 1A, Movie in Additional file 1). In some cases, the Golgi was located within the cell body, as previously reported in migrating cortical interneurons ([20, 24], data not shown). As myosin ii motor activity and F-actin dynamics in the leading process are crucial for centrosomal two-stroke motility, we next used the fluorescence-labeled utrophin actin-binding domain (UTRCH-ABD) and Lifeact as convenient reporters of actin localization [27, 28], in relation to the Golgi apparatus. During neuronal migration, we observed high levels of red fluorescence protein (RFP)-UTRCH-ABD in the neuronal soma and the leading process (Figure 1B, Movie in Additional file 2). RFP-UTRCH-ABD was enriched in the proximal portion of the leading process (also referred to as the cytoplasmic dilation), which widened before somal translocation when the Golgi apparatus translocated forward into this region. We observed a similar relationship between proximal leading process F-actin and the Golgi apparatus when using a teal fluorescent protein (TFP)-Lifeact construct (Figure 1E, Movie in Additional file 3). We quantitated F-actin concentration in relation to the Golgi using a newly developed 4D volumetric analysis algorithm that involved measuring the 3D intensity and volume of actin and the Golgi over time in our imaging sequences. The analyses displayed as adaptive volumetric kymographs indicate that when the Golgi translocated forward, it left the F-actin zone in the vicinity of the cell soma and was located within the boundaries of F-actin in the proximal portion of the leading process (Figure 1C,F). Moreover, F-actin concentration in the immediate vicinity of the Golgi was periodically higher than the average concentration of F-actin throughout the cell (Figure 1D,G). Our laboratory and others have shown that F-actin flows from the proximal to distal leading process during migration [11–13, 15]. 2D intensity plots show the Golgi is localized to the leading process F-actin that flows down the leading process prior to cell soma movement (Movies in Additional files 4 and 5). Taken together, these results show that the two-stroke movement of the Golgi apparatus is similar to that of the centrosome and, like centrosomal translocation, coincides with leading-process F-actin dynamics.

### Myosin ii and F-actin dynamics are required for Golgi apparatus translocation in migrating CGNs

**Figure 2:**
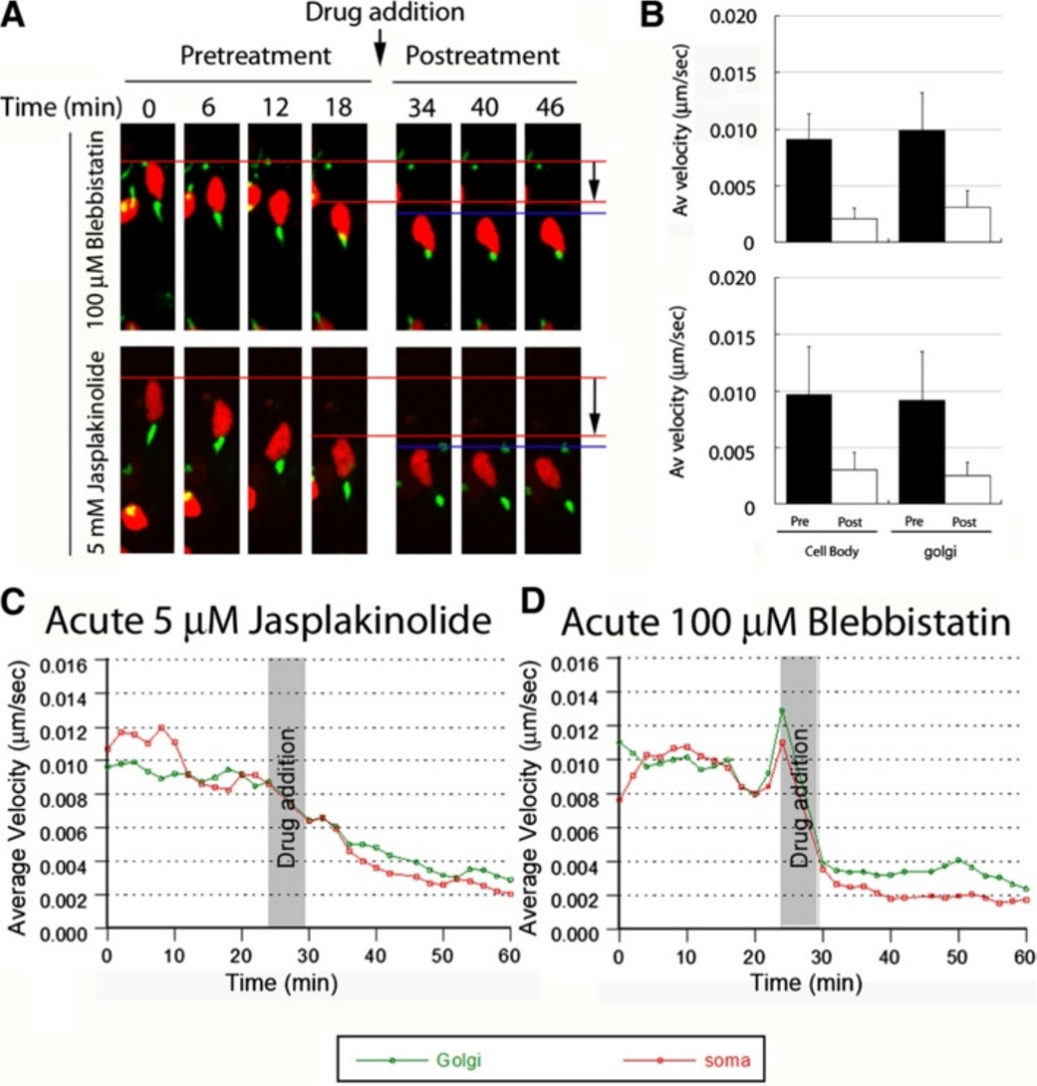
**Myosin ii and F-actin dynamics and motor activity are required for coordinated movement of the Golgi and cell body.** CGNs were transfected to express the Golgi label GalNAcT2-Venus (green) and the nucleus label H2B-mCherry (red). Velocity of the Golgi and soma in migrating neurons was measured by time-lapse imaging. After 24 minutes of migration, 100 μM blebbistatin or 5 μM jasplakinolide were added to the culture, and imaging continued for a further 36 minutes. **(A)** Shows representative time-lapse images. Both drugs potently inhibited forward movement of the Golgi and cell body. **(B)** Cell body and Golgi velocity before and after drug treatment. Upper panel: 100 μM blebbistatin reduced mean cell body velocity from 0.009 ± 0.002 (SD) to 0.002 ± 0.001 μm/sec (n = 36). Golgi mean velocity was reduced from 0.010 ± 0.003 to 0.003 ± 0.001 μm/sec (n = 48). Lower panel: 5 μM jasplakinolide similarly reduced average velocity of the soma and Golgi. Mean cell body velocity was reduced from 0.010 ± 0.003 to 0.004 ± 0.002 μm/sec (n = 28), and mean Golgi velocity was reduced from 0.009 ± 0.002 to 0.004 ± 0.001 μm/sec (n = 39). All changes in velocity were statistically significant (*P* <0.0001, *t*-test). **(C, D)** Reduction of mean velocity of Golgi (green) and cell body (red) after addition of **(C)** 5 μM jasplakinolide (n = 39 Golgi, 28 cell bodies) or **(D)** 100 μM blebbistatin (n = 48 Golgi, 36 cell bodies).

During the first phase of the two-stroke cycle, before somal translocation, leading-process myosin ii motors transport F-actin forward, also moving the centrosome in the direction of migration. Having found that the Golgi apparatus translocates at a time when F-actin accumulates in the CGN leading process, we hypothesized that myosin ii motors and F-actin dynamics power Golgi apparatus movement. We induced expression of H2B-mCherry and GalNAcT2-YFP to label the nucleus and Golgi apparatus, respectively, and used time-lapse imaging to assay their movement before and after addition of 100 μM blebbistatin (which inhibits myosin ii motor activity, [29]) or 5 μM jasplakinolide (which hyperstabilizes actin filaments, [30]). Both agents rapidly reduced Golgi and nuclear motility (Figure 2A,B, Movies in Additional files 6 and 7). Blebbistatin reduced mean nuclear velocity from 0.009 ± 0.002 (SD) μm/sec to 0.002 ± 0.001 μm/sec (n = 36), and mean Golgi apparatus velocity from 0.010 ± 0.003 μm/sec to 0.003 ± 0.001 μm/sec (n = 48). Jasplakinolide reduced mean nuclear velocity from 0.010 ± 0.003 to 0.004 ± 0.002 μm/sec (n = 28) and mean Golgi apparatus velocity from 0.009 ± 0.002 to 0.004 ± 0.001 μm/sec (n = 39). All changes in average velocity due to drug addition were found to be statistically significant (*P* <0.0001, *t*-test). Kinetic analysis of these data revealed that the Golgi apparatus and nucleus ceased movement nearly simultaneously after addition of either agent (Figure 2C,D). Taken together, these results demonstrate that translocation of the Golgi apparatus, much like centrosome translocation [11], requires myosin ii motor activity and F-actin dynamics.

### Myosin ii and F-actin dynamics are required for basal motility of the Golgi apparatus in stationary CGNs

**Figure 3:**
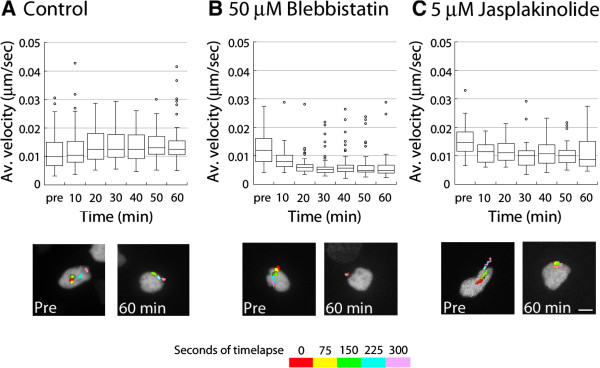
**Myosin ii and F-actin dynamics motor activity are required for basal motility of Golgi apparatus.** Golgi were imaged in non-migrating CGNs for 1 hour in control (untreated), jasplakinolide-treated, and blebbistatin-treated neurons; average Golgi velocity was measured before and at various time points after addition of cytoskeletal drugs. **(A)** In control neurons, Golgi velocity remained stable (mean velocity, 0.013 ± 0.006 (SD) μm/sec, n = 53 to 70 Golgi, depending on time point). Below, motility of the Golgi before treatment and at 60 minutes is supported by representative imaging sequences showing the temporal positions of the Golgi (position of the organelle is shown at every 75 seconds in a different color as indicated by the key). Golgi appear to be moving similarly in both images. **(B)** Golgi velocity diminishes rapidly after treatment with 50 μM blebbistatin. Golgi are motile in the pre-treatment image sequence (mean velocity, 0.013 ± 0.006 μm/sec, n = 52) but almost stationary after 60 minutes of blebbistatin treatment (mean velocity, 0.006 ± 0.005 μm/sec, n = 38, *P* <0.0001, *t*-test). **(C)** Golgi velocity diminishes rapidly after treatment with 5 μM jasplakinolide. The mean velocity was 0.015 ± 0.005 μm/sec (n = 34) before treatment and 0.011 ± 0.005 μm/sec (n = 58, *P* <0.0001, *t*-test) after 60 minutes of jasplakinolide treatment. This is also reflected in the images below, comparing pre-treatment movement to 60-minutes post-treatment.

It was possible that blebbistatin or jasplakinolide might have blocked cell movement rather than exerting an organelle-specific effect. In stationary neurons, the Golgi apparatus undergoes random movements that can be used to assay the forces necessary for Golgi apparatus motility in the absence of cellular movement (i.e., basal motility). To determine whether actin dynamics and myosin ii activity are required for basal Golgi apparatus motility, we monitored Golgi apparatus movement by time-lapse imaging in stationary neurons expressing GalNAcT2-YFP, before and after the addition of blebbistatin or jasplakinolide. Five-minute recordings before drug addition preceded six further 5-minute movies taken every 10 minutes over the course of 1 hour after drug addition. In control (untreated) neurons (n = 52), the Golgi apparatus moved at a mean velocity of 0.013 ± 0.006 μm/sec (Figure 3A, Movie in Additional file 8). Mean velocity was significantly reduced (to 0.006 ± 0.005 μm/sec, n = 38; Figure 3B, Movie in Additional file 9) after 60 minutes of blebbistatin treatment (*P* <0.0001, *t*-test). Jasplakinolide reduced Golgi mean velocity to a lesser extent from 0.015 ± 0.005 to 0.011 ± 0.005 μm/sec after 60 minutes; however, *t*-test of this data showed this to be a significant decrease (*P* <0.0001) (Figure 3C, Movie in Additional file 10). These results established that Golgi apparatus motility is dependent on myosin ii motor activity and F-actin dynamics.

### Translocation of primary cilia displays two-stroke motility and parallels F-actin dynamics

To expand our examination of organelle positioning during the first phase of the two-stroke cycle, we next assessed the motility of primary cilia in migrating CGNs. While mother centriole positioning and primary cilia have been examined in migrating interneurons [31, 32], we found no reports describing a direct examination of the motility of primary cilia during radial migration. The primary cilium, composed of a membrane-docked mother centriole, axonemal microtubules, and a cilia-associated plasma membrane [33–35], differs from the Centrin2-labeled daughter centriole examined in previous imaging studies [11, 18, 19, 21–23], which is primarily a cytoplasmic organelle with limited attachment to cytoskeletal or membrane structures [36]. The primary cilium provides an opportunity to examine the interaction of the actomyosin cytoskeleton with the positioning of discrete membrane and cytoskeletal domains.

**Figure 4:**
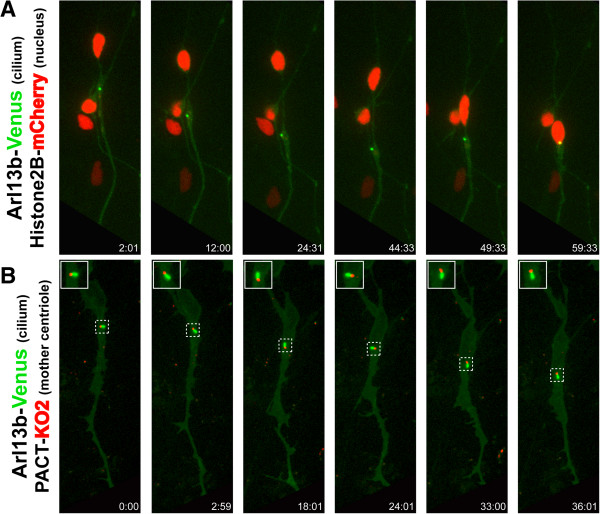
**The primary cilium displays two-stroke motility dynamics.** **(A)** The primary cilium (labeled by Arl13b-Venus, green) translocates before the nucleus (labeled by H2B-mCherry, red) moves forward. **(B)** The primary cilium (green) and mother centriole (labeled with PACT-KO1, red) translocate forward in unison during the two-stroke nucleokinesis cycle.

Time-lapse imaging of CGNs expressing H2B-mCherry and Arl13b tagged with Venus, a Ras-related GTPase essential for cilia function [32, 37, 38], revealed that the primary cilium behaves similarly to the centrosome, entering a dilated region near the proximal portion of the CGN leading process (Figure 4A, Movie in Additional file 11). This imaging probe was validated by comparing the localization of the mother centriole, identified by labeling the pericentrin PACT domain with Kusibira orange fluorophore (KO2), to that of primary cilia in migrating neurons. As expected, in time-lapse imaging the Arl13b-Venus-labeled primary cilium co-migrated with the PACT-KO2-labeled mother centriole as both structures entered the leading process during the two-stroke motility cycle (Figure 4B, Movie in Additional file 12).

**Figure 5:**
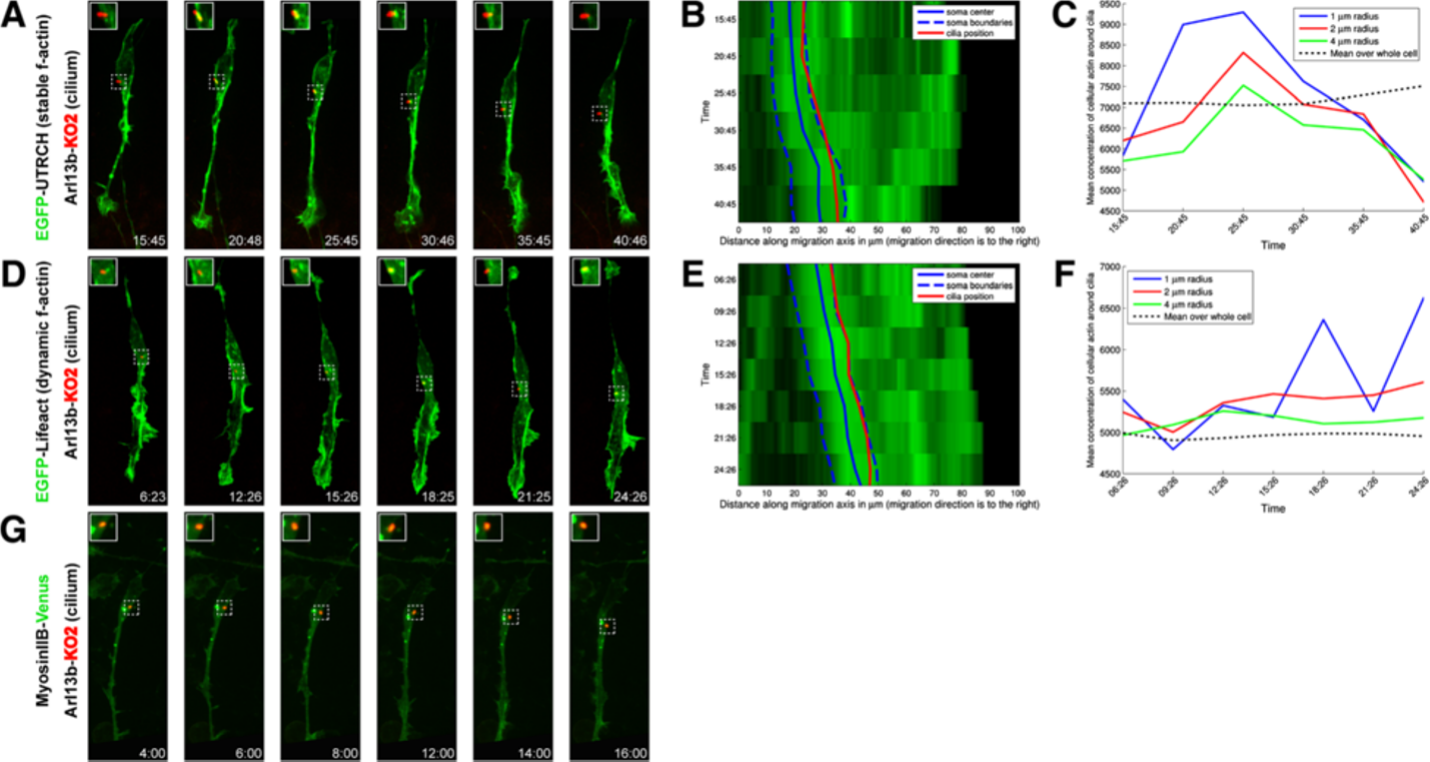
**The primary cilium is embedded in the actomyosin network during the two-stroke motility cycle.** The primary cilium (labeled by Arl13b-KO1, red) is embedded within **(A)** the stable F-actin (labeled by EGFP-UTRCH, green), **(D)** the dynamic F-actin (labeled by EGFP-Lifeact, green), and **(G)** the myosin ii heavy chain-labeled (labeled by MHCiiB-Venus, green) contractile domains in the leading process of living neurons. **(B, E)** Adaptive volumetric kymographs of the sequences shown in Panel A and D (somal boundaries, dashed blue line; soma center, solid blue line; cilium position, red line; EGFP-UTRCH or EGFP-Lifeact, green). **(C, F)** Analysis of mean actin cilium computed within a 1, 2, or 4 μm radius from the center of the cilium (black dashed line represents the average actin concentration throughout the cell). Scale bar, 10 μm. Inset shows the extent of co-localization at each time point. Scale bar, 10 μm.

Given the role of the actomyosin cytoskeleton in the positioning of other organelles during the first step of the two-stroke motility cycle, we examined the relation of primary cilia motility to this structure in the neuronal leading process. Time-lapse imaging of CGNs expressing Arl13b-KO2 and either UTRCH-ABD-EGFP or Lifeact-EGFP and adaptive volumetric kymograph analysis or measurement of local F-actin concentrations revealed enrichment of F-actin near the primary cilia during stages of the migration cycle (Figure 5A–F, Movies in Additional files 13 and 14). The imaging also revealed the primary cilia to be co-localized with a portion of the leading process myosin ii heavy chain-rich domain (as visualized by Venus-MHCiiB; Figure 5G, Movie in Additional file 15). Taken together, our time-lapse imaging studies indicated that neuronal primary cilia participate in the two-stroke nucleokinesis cycle and appear to interact with the actin cytoskeleton during neuronal migration.

### Myosin ii and F-actin dynamics are required for translocation of primary cilia in migrating CGNs

**Figure 6:**
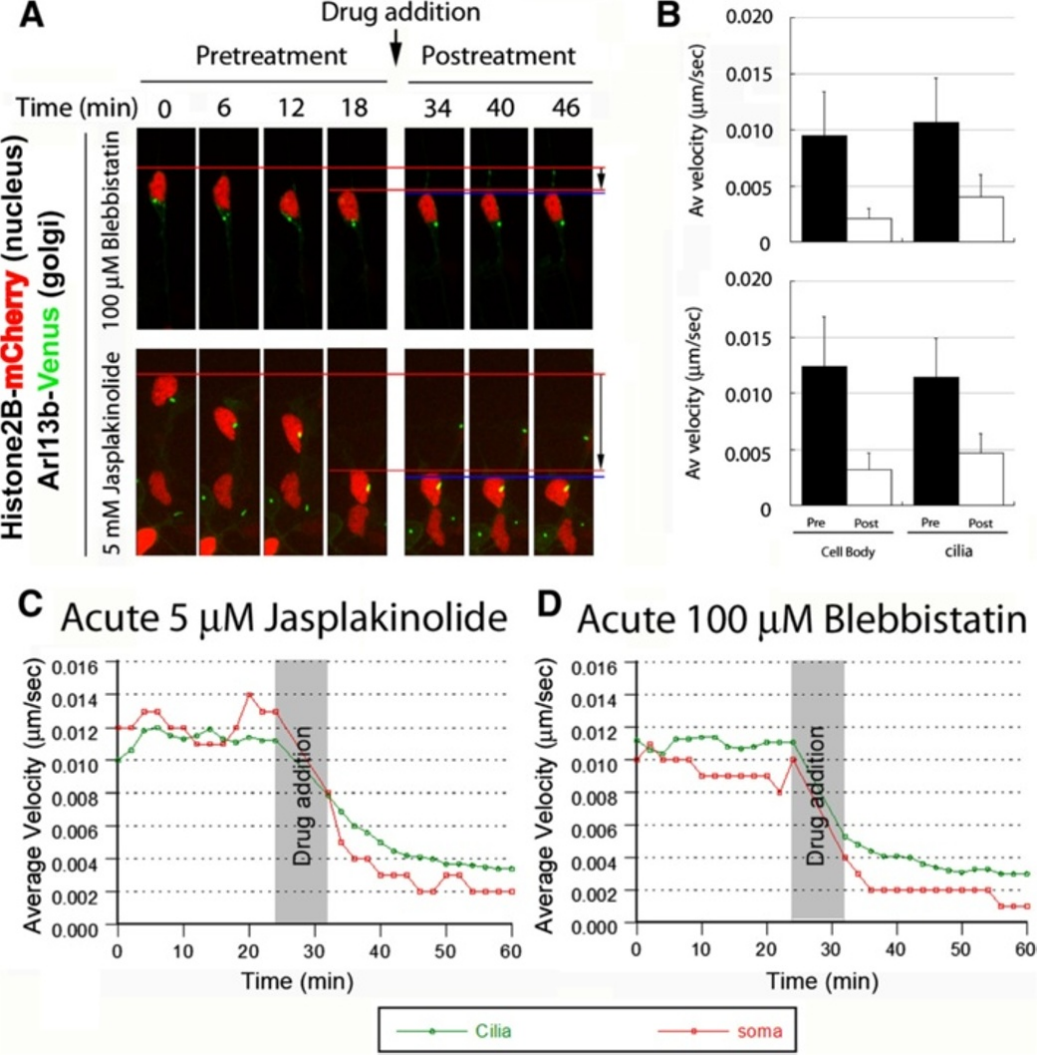
**Myosin ii and F-actin dynamics motor activity are required for coordinated movement of the cilia and cell body.** CGNs were induced to express the cilia label Arl13b-Venus (green) and the nucleus label H2B-mCherry (red), and time-lapse imaging was used to measure velocity of the cilia and soma in migrating neurons. After migration for 18 minutes, 100 μM blebbistatin or 5 μM jasplakinolide were added, and imaging continued for a further 28 minutes. Representative time-lapse images are shown. **(A)** Both drugs potently inhibited forward movement. **(B)** Velocity of the cell body and cilia before and after drug treatment; 100 μM blebbistatin (upper) reduced mean cell body velocity from 0.009 ± 0.004 to 0.002 ± 0.001 μm/sec (n = 191) and reduced mean cilia velocity from 0.011 ± 0.004 to 0.004 ± 0.002 μm/sec (n = 163); 5 μM jasplakinolide (lower) reduced mean cell body velocity from 0.012 ± 0.004 to 0.003 ± 0.002 μm/sec (n = 128) and reduced mean cilia velocity from 0.011 ± 0.004 to 0.005 ± 0.002 μm/sec (n = 143). All changes in velocity were statistically significant (*P* <0.0001, *t*-test). **(C, D)** Decrease in mean velocity at each time point after addition of **(C)** 5 μM jasplakinolide (n = 143 cilia, 128 somas) (see movie) and **(D)** 100 μM blebbistatin n = 163 cilia, 191 somas).

Given the apparent co-incidence of the primary cilia and actomyosin cytoskeleton during CGN migration, we next tested whether actomyosin contractility is responsible for the motility of primary cilia during two-stroke nucleokinesis. Nuclear and primary cilia movement was monitored by time-lapse imaging before and after addition of 100 μM blebbistatin or 5 μM jasplakinolide. As above, both drugs rapidly reduced movement of the organelles (Figure 6A,B, Movies in Additional files 16 and 17). Blebbistatin reduced mean velocity from 0.009 ± 0.004 μm/sec to 0.002 ± 0.001 μm/sec in 191 nuclei and from 0.011 ± 0.004 μm/sec to 0.004 ± 0.002 μm/sec in 163 primary cilia. Jasplakinolide reduced mean velocity from 0.012 ± 0.004 μm/sec to 0.0034 ± 0.002 μm/sec in 128 nuclei and from 0.011 ± 0.004 μm/sec to 0.005 ± 0.002 μm/sec in 143 primary cilia. All reduction in cilia velocity due to drug addition was found to be significant using the *t*-test (*P* <0001). Kinetic analysis revealed that movement of the two organelles was reduced nearly simultaneously by the two agents (Figure 6C,D). We also examined whether the Golgi and primary cilia stop are halted with similar timing in migrating neurons when actomyosin activity is perturbed. The time-lapse panels shown in Additional file 18 show both drugs potently inhibited forward movement of the Golgi, primary cilia, and cell body. Moreover, examination of the microtubule cytoskeleton showed no overt changes in microtubule dynamics or tubulin modification during the acute drug treatments we employed (Additional files 19: Figure S2 and 20: Figure S3). Taken together these results showed for the first time that motility of the primary cilia during two-stroke nucleokinesis requires myosin ii motor activity and F-actin dynamics.

### Myosin ii and F-actin dynamics are required for basal motility of primary cilia in stationary CGNs

**Figure 7:**
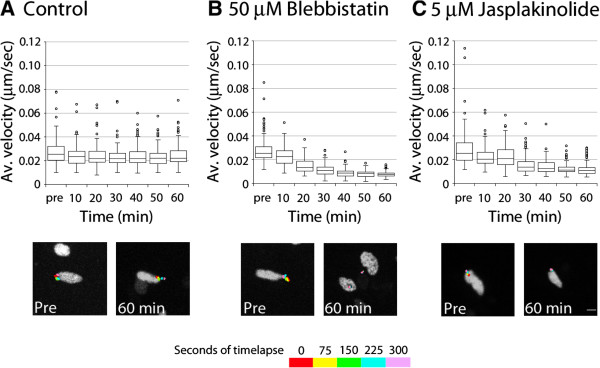
**Myosin ii and F-actin dynamics motor activity is required for basal motility of cilia.** Cilia were imaged for 1 hour in control, jasplakinolide-treated, and blebbistatin-treated non-migrating CGNs. The average velocity was measured before and at various time points after addition of cytoskeletal drugs. **(A)** In control neurons, mean cilia velocity was similar at all time points (mean velocity at each time point ranged from 0.028 to 0.024 (±0.010) μm/sec, n = 104 to 144 cilia per time point). Below, motility of the cilia before treatment and at 60 minutes is supported by representative imaging sequences showing the temporal positions of the cilia (position of the organelle is shown at every 75 seconds in a different color as indicated by the key). Cilia appear to be moving similarly in both images. **(B)** Cilia velocity rapidly diminished after treatment with 50 μM blebbistatin; the cilia were motile before treatment (mean velocity, 0.028 ± 0.011 μm/sec, n = 133 cilia) and almost stationary after 60 minutes of treatment (mean velocity, 0.008 ± 0.003 μm/sec, n = 124 cilia). **(C)** Cilia velocity also rapidly diminished after treatment with 5 μM jasplakinolide; the cilia were motile before treatment (mean velocity, 0.030 ± 0.015 μm/sec, n = 122 cilia), but mean velocity was reduced to 0.012 ± 0.005 μm/sec (n = 138 cilia) after 60 minutes of treatment.

We next assessed the contribution of the actomyosin cytoskeleton to the basal motility of primary cilia in non-migrating CGNs. Using time-lapse imaging, we monitored primary cilia movement in stationary neurons expressing Arl13b-Venus, before and after the addition of blebbistatin or jasplakinolide. The primary cilia moved rapidly in control CGNs (mean velocity, 0.025 ± 0.010 μm/sec) (Figure 7A, Movie in Additional file 21). Mean velocity was significantly reduced (*P* <0.0001, *t*-test) after 60 minutes of blebbistatin treatment (from 0.028 ± 0.011 to 0.008 ± 0.003 μm/sec, n = 124 cilia; Figure 7B, Movie in Additional file 22) and by jasplakinolide treatment (from 0.030 ± 0.015 [n = 122] to 0.012 ± 0.005 μm/sec [n = 138], *P* <0.0001, *t*-test; Figure 7C, Movie in Additional file 23). These results established that the motility of primary cilia is dependent on myosin ii motor activity and F-actin dynamics.

### Jam-C and Cadm3 cell-cell contacts are enriched in the leading process base and soma near actin accumulation sites

Although studies have begun to examine the location of adhesive contacts and trafficking of adhesion receptors in migrating neurons (reviewed in [39, 40]), a significant remaining challenge is to unravel the relationship between the sites of cell contact and the underlying cytoskeletal elements that control adhesion in migrating neurons. Recent findings in non-neuronal cell motility suggest that myosin ii motors and the actin cytoskeleton recruited to sites of cell contact play a direct role in strengthening substrate adhesion in the leading portion of migrating cells and that tension generated by myosin ii-based adhesion forward of the nucleus is required for efficient cell motility [41–44]. Given the expanding known functions of leading-process actomyosin in neurons [11–13, 15] and the role of actomyosin in controlling adhesion in non-neuronal cells, we next tested whether leading-process actomyosin regulates adhesion dynamics in migrating neurons.

**Figure 8:**
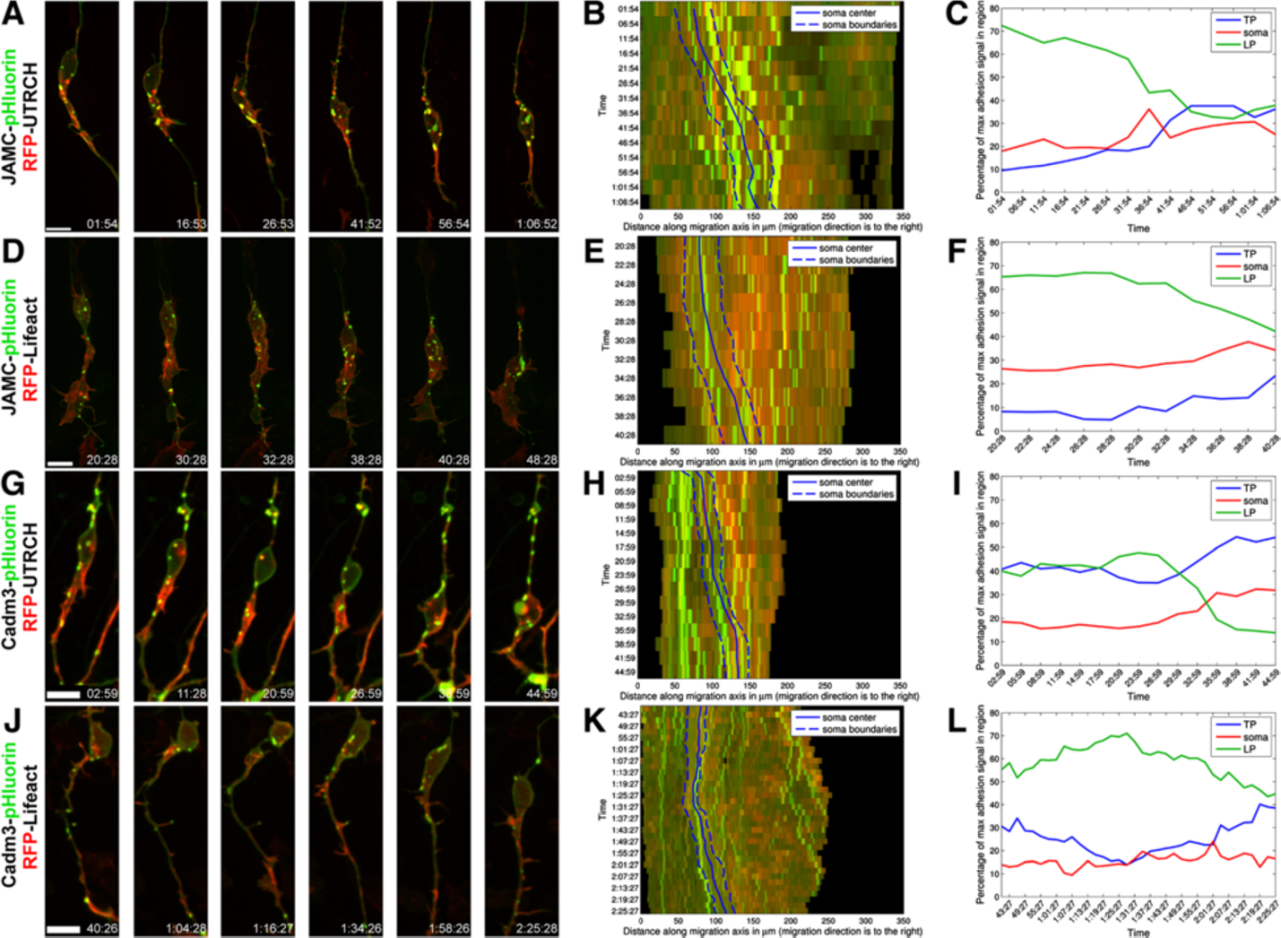
**JAM-C and Cadm3 adhesions are located in the soma and proximal leading process.** **(A, D)** CGNs were electroporated with expression vectors encoding JAM-C-pHluorin and either RFP-UTRCH or RFP-Lifeact. During early migration, JAM-C cell adhesions are observed in the leading process and then accumulate in the trailing process as the soma translocates past the initial adhesion sites in the leading process. JAM-C adhesions in the leading process appear in regions enriched in stable F-actin (labeled by RFP-UTRCH) and dynamic F-actin (labeled by RFP-Lifeact). **(B, E)** Adaptive volumetric kymographs of the sequences showing the relation of JAM-C adhesions to actin within the soma and leading or trailing processes for the sequences are shown in panel A or D (somal boundaries, dashed blue line; soma center, solid blue line; RFP-UTRCH or RFP-Lifeact, green). **(C, F)** Analysis of percentage of adhesion signal in the soma and leading or trailing process in migrating neurons expressing JAM-C (green, leading process; red, soma; blue, trailing process). **(G, J)** CGNs were electroporated with expression vectors encoding Cadm3-pHluorin and either RFP-UTRCH or RFP-Lifeact. During migration, Cadm3 cell adhesions are observed in the leading process and then accumulate in the trailing process as the soma translocates; adhesions in the leading process appear in regions enriched in stable F-actin (labeled by RFP-UTRCH) and dynamic F-actin (labeled by RFP-Lifeact). **(H, K)** Adaptive volumetric kymographs of the sequences showing the relation of Cadm3 adhesions to actin within the soma and leading or trailing processes for the sequences are shown in panel A or D (somal boundaries, dashed blue line; soma center, solid blue line; RFP-UTRCH or RFP-Lifeact, green). (**I, L**) Analysis of percentage of adhesion signal in the soma and leading or trailing process in migrating neurons expressing Cadm3 (green, leading process; red, soma; blue, trailing process). Scale bar, 10 μm.

As the first step, we used pHluorin-based cell contact imaging probes developed in our laboratory to examine the spatial relationship of neuronal adhesive contacts to the actin cytoskeleton. The probes consist of super ecliptic pHluorin [45] fused to the extracellular domain of adhesion receptors, including JAM-C and Cadm3 [46, 47]. As pHluorin fluoresces only at a neutral pH, these probes, like those at the cell surface, allow direct visualization of the dynamic recruitment or engagement of adhesion receptors to the surface of migrating neurons, ultimately reporting the presence of adhesion receptors in living cells with a profile similar to the endogenous receptors detected by fixed-cell antibody stains [46, 47]. Time-lapse imaging of CGNs expressing pHluorin-tagged JAM-C, an immunoglobulin superfamily adhesion molecule essential for CGN migration [47], and either UTRCH-ABD-EGFP or Lifeact-EGFP, revealed surface JAM-C at punctate junctions within the leading process and in larger adhesion plaques within the neuronal soma (Figure 8A,D, Movies in Additional files 24, 25, 26 and 27). Adaptive volumetric kymographs and analysis of the percentage of JAM-C fluorescence in the leading-, trailing-process, or soma revealed that JAM-C adhesions were initially enriched in the F-actin-rich regions in the proximal leading process, where actomyosin activity is known to power nucleokinesis, and in the neuronal soma. As the soma translocated forward, these leading-process junctions coalesced into larger plaques in the soma or trailing process. Surprisingly, the soma translocated past adhesion plaques originally located in the leading process as fluorescence-associated plaques subsequently coalesced within the trailing process.

We examined the localization of pHluorin-tagged Cadm3, an immunoglobulin superfamily adhesion molecule expressed in CGNs at neuron-neuron and neuron-glial contacts [48], to determine whether the observed dynamics of JAM-C were unique or applied to other adhesion molecules. Time-lapse imaging of CGNs expressing Cadm3 pHluorin and either UTRCH-ABD-EGFP or Lifeact-EGFP revealed surface Cadm3 at punctate junctions within the leading process and in larger adhesion plaques within the neuronal soma (Figure 8C,D, Movies in Additional files 28, 29, 30 and 31). Adaptive volumetric kymographs and analysis of the percentage of Cadm3-pHluorin fluorescence in the leading-, trailing-process, or soma revealed the Cadm3-pHluorin-labeled puncta also appeared in F-actin-enriched regions of the proximal leading process and soma. Similar to JAM-C, Cadm3-pHluorin levels were initially high in the leading process and then coalesced into the trailing process as the cell body translocated past adhesions that were initially located in the leading process. Taken together these results suggest that the main sites of JAM-C and Cadm3 adhesive cell contact reside within the soma and the proximal portion of the leading process in migrating CGNs, near regions of F-actin enrichment.

### Myosin ii and F-actin dynamics are required for adhesion dynamics in migrating CGNs

**Figure 9:**
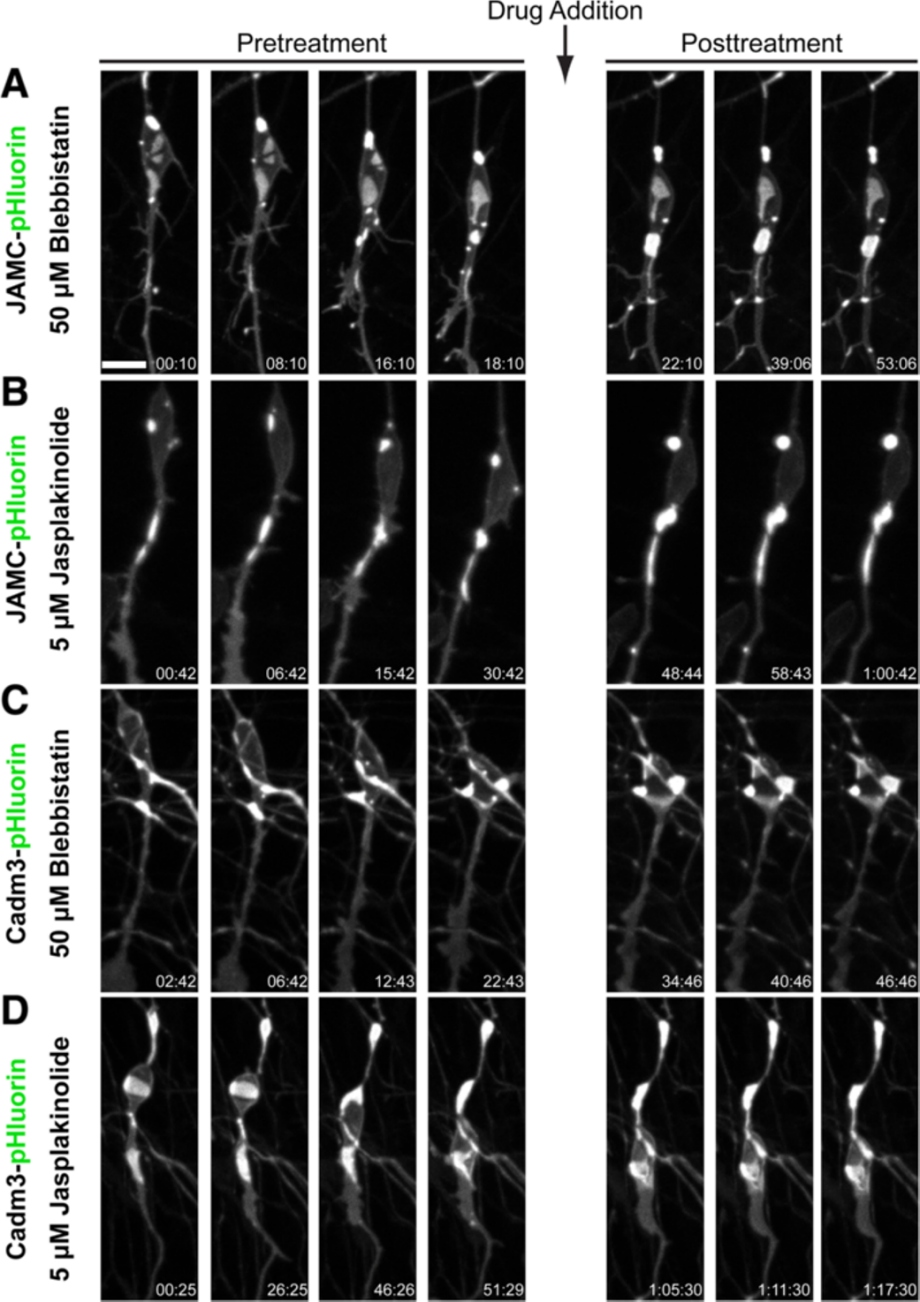
**Myosin ii and F-actin dynamics motor activity are required for JAM-C and Cadm3 adhesion dynamics in migrating neurons.** CGNs were transfected with expression vectors encoding JAM-C-pHluorin **(A, B)** or Cadm3-pHluorin **(C, D)**. Time-lapse imaging was used to monitor cell surface adhesion dynamics in migrating cells. After cells were allowed to migrate for 18 to 50 minutes, 50 μM blebbistatin or 5 μM jasplakinolide were added to the culture and imaging continued for a further 28 minutes. Addition of either drug potently inhibited forward movement, JAM-C dynamics, and Cadm3 adhesions. Scale bar, 10 μm.

Given the apparent localization of JAM-C and Cadm3 contacts within regions of F-actin enrichment, we directly tested the hypothesis that leading-process actomyosin regulates the dynamics of adhesion molecules in migrating neurons by monitoring CGN adhesions before and after addition of blebbistatin or jasplakinolide. Imaging of CGNs nucleofected to express either JAM-C-pHluorin or Cadm3-pHluorin revealed that, before addition of cytoskeletal drugs, JAM-C- and Cadm3-labeled adhesions underwent remodeling as they migrated through the imaging field (Figure 9, pre-treatment frames, Movies in Additional files 32, 33, 34 and 35). Interestingly, after addition of blebbistatin or jasplakinolide, the dynamics of JAM-C and Cadm3 adhesions were curtailed, the adhesions appeared static (Figure 9, post-treatment frames, Movies in Additional files 32, 33, 34 and 35), and few new adhesions appeared.

**Figure 10:**
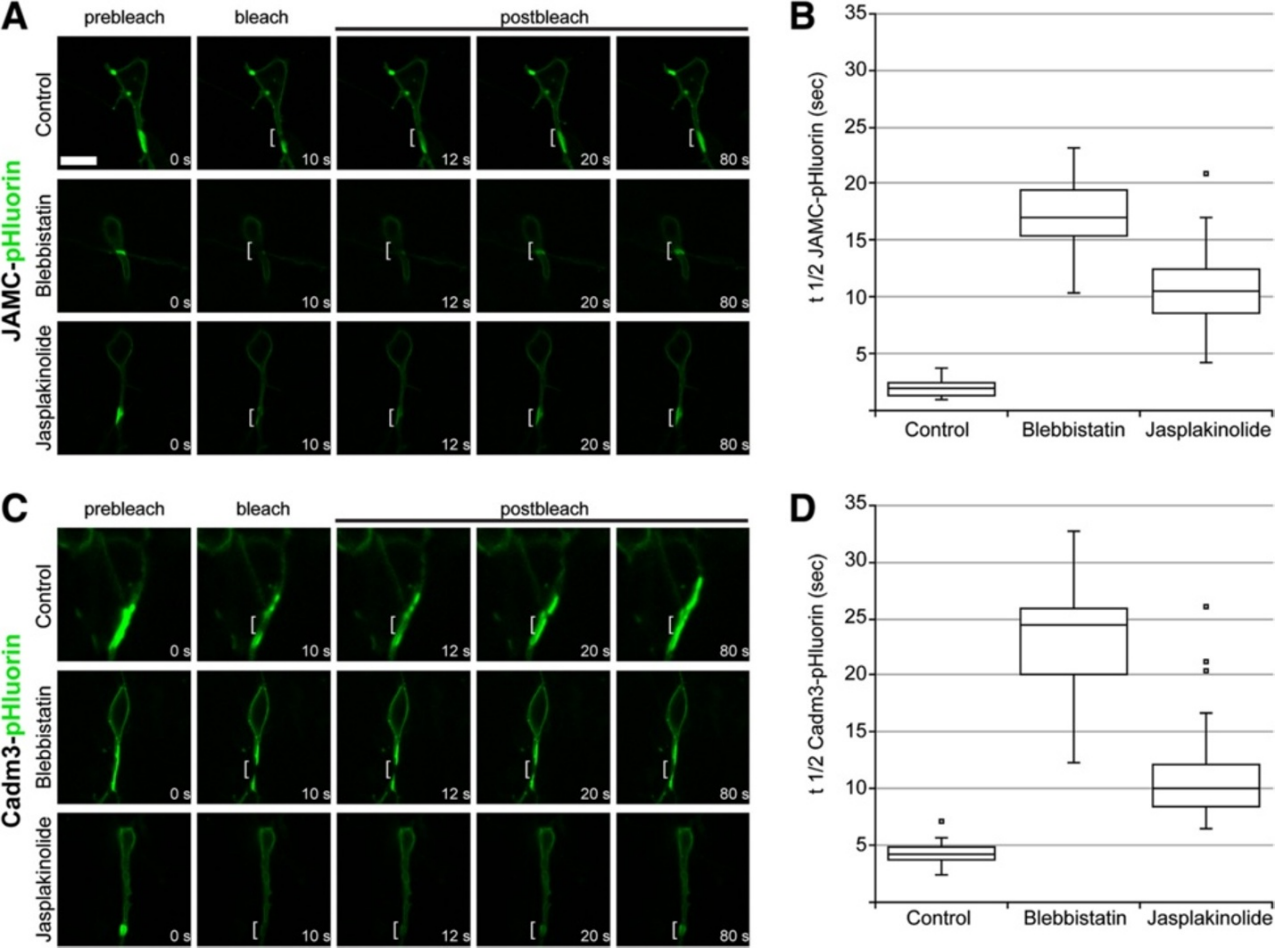
**FRAP analysis of JAM-C and Cadm3 adhesion dynamics in migrating neurons.** CGNs were transfected with expression vectors encoding JAM-C-pHluorin or Cadm3-pHluorin, and FRAP was used to track cell surface adhesion receptor diffusion and recruitment. Blebbistatin (50 μM) or jasplakinolide (5 μM) potently retarded the recruitment of JAM-C **(A, B)** and Cadm3 **(C, D)** to photobleached adhesion sites within the neuronal leading process. Blebbistatin and jasplakinolide increased the t1/2 of recovery of JAM-C-pHluorin fluorescence from 1.94 ± 0.75 sec to 17.34 ± 3.16 sec (n = 30 cells; *P* = 9.64 × 10^-22^, *t*-test) and to 10.63 ± 3.88 sec (n = 25 cells; *P* = 1.07 × 10^-11^, *t*-test), respectively. Blebbistatin and jasplakinolide increased the t1/2 of Cadm3-pHluorin fluorescence recovery from 4.26 ± 0.98 sec to 23.28 ± 4.77 sec (n = 30 cells; *P* = 1.83 × 10^-19^, *t*-test) and to 11.19 ± 4.52 sec (n = 30 cells, *P* = 1.69 × 10^-8^, *t*-test), respectively. Scale bar = 10 μm. A bracket highlights the photobleached region of interest in each representative image.

We next quantified the apparent reduction of JAM-C and Cadm3 movement by using fluorescence recovery after photobleaching (FRAP). We focused on the proximal leading process and soma, which displayed the greatest number of cell contacts in migrating neurons. In a set of FRAP experiments, JAM-C-pHluorin and Cadm3-pHluorin were photobleached in regions of interest in the proximal leading process of neurons treated with 100 μM blebbistatin or 5 μM jasplakinolide (concentrations that inhibited migration and organelle motility). The mean fluorescence recovery time (t1/2) of JAM-C was very short (1.94 ± 0.75 [SD] sec, n = 30 cells; Figure 10A). Blebbistatin treatment increased the recovery time of JAM-C pHluorin by nearly 9-fold (t1/2 = 17.34 ± 3.16 sec, n = 30 cells) and jasplakinolide increased it by 5-fold (t1/2 = 10.63 ± 3.88 sec, n = 25 cells; Figure 10A). The recovery time of Cadm3 fluorescence was also rapid (mean, 4.26 ± 0.98 sec, n = 30 cells; Figure 10B). Blebbistatin treatment increased it by 5-fold (t1/2 = 23.28 ± 4.77 sec, n = 30 cells) and jasplakinolide increased it by nearly 3-fold (t1/2 = 11.19 ± 4.52 sec, n = 30 cells; Figure 10A). Interestingly, this result is similar to that in a recent report demonstrating that actomyosin flow is required for diffusion of guidance receptors within the CGN leading process [49]. Taken together, these results show that myosin ii motor activity and F-actin dynamics are required for adhesion receptor dynamics in migrating neurons. Moreover, the results of FRAP analysis suggest that adhesion receptors are static in the absence of myosin ii motor activity and F-actin dynamics because of reduced recruitment of fresh JAM-C and Cadm3 to sites of cell-cell contact.

## Conclusions

Studies in our laboratory previously showed that leading-process actomyosin flow orchestrates the saltatory movement of CGNs along glial fibers by controlling positioning of the centrosome and soma during the two-stroke nucleokinesis cycle [11]. However, the overall function of actomyosin contractility in relation to the polarity of the two-stroke motility cycle displayed by migrating neurons remains to be elucidated. Here, we have shown that leading-process actomyosin globally coordinates the positional polarity of multiple organelles before nucleokinesis and regulates adhesive traction forces within the leading process of the migrating neuron. We would like to note that, while the preponderance of actomyosin in the CGN leading process suggests this is the main site for actin and myosin coordination of these events, we cannot discount that minor populations of actin in other regions of the cell may also contribute until more focal techniques are applied to CGNs.

The orientation of the Golgi apparatus defines the axis of polarity and the vectorial flow of membrane traffic in a variety of cell types. The Golgi apparatus reorients toward the “leading edge” in epithelial or astrocyte monolayers induced to migrate in scratch assays [50, 51], reorients toward immune synapses in immune cells [52, 53], and localizes to axons or dendrites at various stages of neuronal polarization [54–57]. Golgi polarization during neuronal migration was first observed in ultrastructural studies [58, 59] and later confirmed in inhibitory rat cortical interneurons [17] and tangentially migrating mouse MGE neurons by using specific Golgi markers [20]. Using fixed MGE cultures, Bellion et al. showed the Golgi to be located within the dilated region of the MGE leading process before nuclear translocation [20], a finding recently confirmed by *in vivo* time lapse imaging [24]. However, the two-stroke motility of this organelle has not been examined in radially migrating neurons and the cytoskeletal components that control its motility have not been identified. Time-lapse imaging of CGNs shows that not only does the Golgi enter the leading process during the first stroke of the nucleokinesis cycle but it also moves with timing and velocity similar to that of the centrosome.

We also showed that F-actin is enriched in the CGN leading process when the Golgi participates in the first step of the two-stroke nucleokinesis cycle, suggesting that the leading-process actomyosin flow responsible for CGN centrosome positioning plays a role in Golgi motility. Direct manipulation of the actomyosin cytoskeleton by blebbistatin and jasplakinolide treatment revealed that myosin ii motor activity and F-actin dynamics are required for Golgi positioning before nuclear translocation. This result was surprising, as actin-based motors like myosin ii [60] and myosin vi [61] have been shown only to play roles in Golgi membrane fusion and fission, not positioning. In most models, cytoplasmic dynein is thought to be the predominant motor regulating Golgi positioning by directing Golgi membranes to the vicinity of the centrosome [62]. Interestingly, Zmuda and Rivas reported that an intact actin cytoskeleton is required for re-alignment of the Golgi within the CGN cell body as CGNs initiate parallel fibers axons prior to migration [55]. Thus, the actin cytoskeleton and myosin ii motors are likely to play an important role in the positioning of the Golgi apparatus, a role that is distinct from the centrosome-directing activity of cytoplasmic dynein.

Primary cilia have recently been reported to transduce crucial signaling, for example, sonic hedgehog and sdf-1-based signaling, that regulates neuron migration in the developing cerebral cortex [31, 32]. Interestingly, preliminary imaging studies have described the primary cilia as motile in cortical interneurons. The dynamics of primary cilia have also not been examined in radially migrating neurons, nor have the cytoskeletal components required for the motility of this organelle been reported in any cell type. In migrating CGNs, primary cilia are localized largely in the proximal portion of the leading process; they move toward the future direction of migration during the first step of the two-stroke nucleokinesis cycle. Imaging of the F-actin cytoskeleton and myosin ii motors demonstrated that the actomyosin cytoskeleton accumulates with the primary cilia during the two-stroke cycle. Myosin ii motor activity and F-actin dynamics are also required for motility of the primary cilia, as blebbistatin and jasplakinolide block the movement of cilia in both migrating and stationary neurons. These results expand the role of the actin cytoskeleton in regulating aspects of cilium biology (reviewed in [34, 63]).

A recent functional genomics screen identified a number of actin regulatory proteins that control the length of primary cilia [64]. While the role of the actin cytoskeleton in regulating the positioning of primary cilia has not been studied in any system, there is compelling evidence that actomyosin regulates the positioning of motile cilia without affecting their beating. Myosin ii motors and actin biochemically fractionate with purified basal body preparations from heavily ciliated cells [65] and appear to accumulate at the base of motile cilia in ultrastructural localization studies [65, 66], much as we observed for CGN primary cilia. Moreover, basal bodies associate with actomyosin before they migrate to the apical domain of polarized epithelial cells [66], and treatment with drugs that inhibit myosin ii motors prevents the docking of the basal body with the cortical actin cytoskeleton [67, 68]. Our findings suggest that the actomyosin association with primary cilia may represent a conserved feature of primary and motile cilia that orients their direction of motility during neuron migration.

Recent advances have revealed a diversity of mechanisms that regulate the adhesion of neurons to their migration substrates; these include adhesion receptor endocytosis, Reelin regulation of pro-adhesive small GTPases, phosphorylation of GAP junctional proteins, and ubiquitin proteasome degradation of polarity proteins [47, 69–75]. Despite these advances, there remains much to be learned about how adhesion dynamics in the two-stroke motility cycle are controlled in live, migrating neurons. Most studies have examined adhesion receptor localization in fixed cells or have not specifically examined cell surface adhesion receptors. Our pHluorin-based imaging probes allowed us to directly examine the cell surface adhesion dynamics at CGN-Bergmann glial junctions in migrating CGNs. JAM-C and Cadm3 formed punctate junctions in the base of the leading process and larger plaques in the soma, as previously described in ultrastructural studies [58, 59] and more recent studies of the localization of astrotactin1 and β1 integrin adhesion receptors [69, 72]. In our study, before somal movement, adhesions formed within actin-rich regions at the base of the leading process and then coalesced into larger plaques within the soma. Previous models of adhesion disassembly during neuronal migration proposed that nucleokinesis and somal translocation would occur simultaneously with adhesion disassembly at the rear of the soma of a migrating neuron [69, 72]. Surprisingly, we observed the soma to slide past many adhesions before these finally disassembled in the CGN trailing process, suggesting that traction forces generated by adhesions within the leading process are more important for somal translocation than their disassembly in the trailing aspect of the neuron. Finally, the actomyosin cytoskeleton in the vicinity of CGN junctions appears to regulate adhesion dynamics, as inhibition of myosin ii motors and slowed F-actin dynamics rendered JAM-C and Cadm3 adhesions static and appeared to block their assembly, disassembly, and movement within the plasma membrane (a finding confirmed and quantified by FRAP). While no previous study has linked the actomyosin cytoskeleton to control of adhesion dynamics in migrating neurons, these results are consistent with recent advances in general models of cell motility. In fibroblasts and epithelial cells, myosin ii bundling of actin filaments and actin polymerization controls adhesion morphology [41–44, 76]. In these systems, nascent adhesions form within actin-rich regions of lamellipodia mature as myosin ii bundles actin filaments within the lamella just forward of the nucleus and disassemble due to actomyosin activity in the rear of the cell. Our results, together with previous findings about leading-process actomyosin flow [11, 12], suggest that the base of the leading process is similar to the lamella of epithelial cells and fibroblasts, as all are actomyosin-rich regions forward of the nucleus that support adhesion dynamics necessary for efficient cell migration.

How does actomyosin coordinate the events that are linked to initial steps of the two-stroke motility cycle prior to nuclear movement? The work of our lab and others shows not only that actomyosin accumulates in the leading process of migrating neurons but also that it flows down the leading process in a myosin ii-dependent fashion. Moreover, specific inactivation of leading-process actomyosin motors halts neuronal migration [12]. It is likely that either direct or indirect linkage between the cortical actomyosin cytoskeleton and organelles or adhesion molecules are necessary to harness actomyosin flow and drive the organelle or surface receptor movements that occur during migration (Additional file 36: Figure S4). When considering actomyosin flow, the movement of the primary cilia and adhesion receptors likely represent the most local activity of the actomyosin cytoskeleton as they occur within the plane of the cell membrane closest to the actomyosin rich cortex. In the future, it will be of great interest to identify the molecules that link organelle movement to actomyosin flow in migrating neurons.

Taken together, our findings show that asymmetric organelle positioning, membrane organization, and adhesion formation define the polarity of nucleokinesis in radially migrating neurons, much as in classical cell polarity models (e.g., epithelial apicobasal polarity or front-to-back polarity in migrating fibroblasts). In polarized epithelia and fibroblasts, myosin ii motor activity executes cell polarity programs after polarity is initiated [77, 78]. Similarly, our results demonstrate that myosin ii activity is required to power many key events linked to polarity during nucleokinesis. Overall, we propose that myosin ii no longer be viewed only as a motor that powers cytoskeletal arrangement for migration; instead, we propose that it be viewed as broadly coordinating the overall polarity of the migrating neuron. In the future, it will be of great interest to determine how extrinsic factors that instruct leading-process extension and ultimately the direction of migration contribute to the asymmetric recruitment of actomyosin flow necessary for polarized organelle positioning and adhesion formation. It will also be of interest to determine whether key polarity proteins, such as the partitioning defective complex, regulate Golgi or cilia positioning and leading process adhesive traction through an interaction with myosin ii motors.

## Methods

### Animals

All mice in this study were housed, bred, and used according to procedures and guidelines approved by Institutional Animal Care and Use Committee at St. Jude Children’s Research Hospital (protocol number = 483).

### Preparation and nucleofection of CGNs

CGNs were prepared as previously described (Hatten, 1985). Briefly, cerebella were dissected from the brains of P7 C57BL6 mice, the pia was peeled away, and cerebellar tissue was treated with trypsin and triturated using a fine-bore fire-polished Pasteur pipettes. A single-cell suspension in CMF-PBS was layered onto a 60% to 35% Percoll gradient, and separated by centrifugation. The cellular fraction at the interphase was isolated (routinely contained 95% CGNs and 5% glia) and transfected by nucleofection. For imaging experiments, CGNs were nucleofected with 1 to 20 μg of pCIG2 expression vector encoding fluorescence-labeled cytoskeletal proteins by using the Amaxa Mouse Neuron kit with A030 or O-005 program on the Amaxa Nucleofector II system (Lonza). Nucleofected CGNs were plated in glass-bottomed movie dishes (Mattek) coated with poly-L-ornithine and Matrigel at a low concentration to facilitate attachment of neurons to glial processes.

### Plasmid vectors

All cDNAs encoding fluorescent fusion proteins were subcloned into the pCIG2 expression vector [79]. pCIG2 H2B-mCherry and pCIG2 JAM-C-pHluorin have been previously described [47]. Franck Polleux provided pCIG2 Lifeact-RFP and pCIG2 Lifeact-EGFP. Graham Warren provided the GalNAcT2-YFP cDNA. Robert Adelstein provided the MHCiiB cDNA. The Arl13b-Venus, Arl13b-KO2, PACT-KO, Cadm3-pHluorin, TFP-UTRCH-ABD, and TFP-Lifeact cDNAs were commercially synthesized and subcloned into pCIG2 by Genscript (Piscataway, NJ, USA).

### Drug treatment experiments

For myosin ii inhibition or F-actin stabilization experiments, cells were imaged before drug addition, the indicated amount of blebbistatin or jasplakinolide was added to the bath, and the cultures were then imaged for an additional time. For acute migration experiments, migrating cells were imaged for 24 minutes (at 2 minute intervals), the indicated drugs were added to the bath, and the cultures were imaged for a further 36 minutes (total length of movie – 60 min, but time points 26 to 32 minutes were not included in analysis to avoid images that were out of focus or movement of cells caused by drug addition). For measurement of basal organelle movement, cells were imaged for 5 minutes at 15-second intervals. Cytoskeletal drugs were then added to the bath and movement was imaged every 10 minutes over the next hour using the same imaging conditions (5 minute movies with 15-second intervals) to examine the changes in organelle motility over time due to drug addition. All blebbistatin-sensitive organelle positioning events were also examined using Kusibira orange labeled imaging probes (e.g., Arl13, Centrin2, and GalNacT2) that were illuminated with 560 nm wavelength light to confirm reductions in organelle velocities were not due to phototoxicity sometimes associated with imaging of blebbistatin with intense blue wavelength light (data not shown). Blebbistatin is frequently used at concentrations between 25 to 100 μM (all of which have been tested on migrating CGNs) and even at 50 to 100 μM concentrations it is specific for inhibiting the related skeletal muscle and non-muscle myosin motors without affecting smooth muscle myosin, Myo 1b, MyoVa, or MyoX [29].

### Imaging and analysis

CGN cultures were imaged with a Marianas Spinning Disk Confocal Microscope (Intelligent Imaging Innovations) comprising a Zeiss Axio Observer microscope equipped with 40×/1.0 NA (oil immersion) and 63×/1.4 NA (oil immersion) PlanApochromat objectives. An Ultraview CSUX1 confocal head with 440 to 514 nm or 488/561 nm excitation filters and ImageEM-intensified CCD camera (Hamamatsu) were used for high-resolution imaging. Z-stacks (10 μm thick, 13 sections per stack) were collected over the duration and time-intervals indicated. Movies were captured, processed, and analyzed using Slidebook software (Intelligent Imaging innovations). Generally, all image analysis was obtained using the manual tracking protocol tool in Slidebook to measure the average velocities of the organelles. Cells were only included into the study if they showed good health throughout the length of the movie, and the organelles of interest were visible over at least five consecutive frames. In acute drug treatment movies, neurons that moved more than two cell body lengths were considered to be migrating neurons.

### Adaptive volumetric kymographs

Kymographs for each time sequence were generated by accumulating the fluorescence of the cell within its 3D segmentation boundaries. We refer to these as “volumetric kymographs” to differentiate them from the typical kymographs that are generated from 2D images. Assuming an accurate 3D segmentation of the cell, this approach provides a more accurate estimate of the fluorescent content of the cell because it takes into account the shape of the cell in the z-dimension, as opposed to summing or computing the max over all slices at each x-y location.

The 3D cell segmentation for a given cell was computed by manually selecting an intensity threshold for that cell and keeping only those voxels that are connected to the soma. In many cases, segmentation “walls” were necessary to prevent the segmentation from bleeding into an adjacent cell in the image. These walls acted as lines of zero intensity in the volume and were drawn manually in the sequence using a custom software program wherever the intensity-based segmentation captured part of a neighboring cell.

Once the 3D segmentation was computed, the volumetric kymograph was computed by analyzing cross-sections of the segmented cell perpendicular to a predefined vector. This vector was defined as the direction from the soma to the tip of the leading process in the first frame of the sequence, which approximates the cell’s direction of migration. Cross-sections of the cell were computed at a distance of one x-y pixel apart along the vector. Examples of these cross-sections can be seen in Supplemental Movies 4 and 5, where five such cross-sections are shown for illustration (but many more were computed). The volumetric kymograph was then generated by computing either the sum or the max of the pixel fluorescent intensities in each cross-section, resulting in a fluorescence signal along the length of the cell. Examples of these signals for the red and green channels are also shown in Supplemental Movies 4 and 5, plotted above the vector line.

### Mean concentration analysis

For image sequences containing Golgi or cilia, the mean concentration of actin was computed in the vicinity of these objects. This was done by first marking the position of the object of interest at each time-point in the sequence and then defining a cylindrical region around the object of a desired radius, where the axis of the cylinder was perpendicular to the z-axis. The intersection of the segmented cell volume and the cylindrical volume was used as the region in which to accumulate actin concentration around the object, and the mean of the actin fluorescent intensity in that region was computed at each time-point. The mean of the actin fluorescence over the entire 3D segmented cell region was also computed as a baseline for comparison. Radii of 1, 2, and 4 μm were used in the computation to assess the actin concentration around the object at different scales.

### FRAP analysis of migrating CGNs

CGNs were nucleofected for 18 hours with expression vectors encoding Jam-C-pHluorin or Cadm3-pHluorin and RFP-UTRCH-ABD. Cultures were then imaged with the Marianas confocal microscope described above, equipped with a Vector high-speed x,y scanner (Intelligent Imaging Innovations) fitted to emit 488-nm light for photobleaching pHluorin-labeled adhesion receptors. Regions of interest were photobleached using 30% laser power and a 20 ms dwell time, and images were acquired at 2-second intervals after bleaching. The half-life (t1/2) of Jam-C-pHluorin or Cadm3-pHluorin and mobile cell fraction were calculated using Slidebook.

## Electronic supplementary material

Additional file 1: Movie 1: The Golgi apparatus (labeled with GalNAcT2-YFP, yellow) enters a dilated region near the proximal portion of the leading process before nuclear translocation (nucleus labeled with CFP-NLS, teal). Images were acquired every minute and 40 seconds. Displayed in Figure 1A. Total elapsed time is 26 minutes. Time is displayed as hours:minutes:seconds. Scale bar, 10 μm. (MOV 3 MB)

Additional file 2: Movie 2: Stable F-actin (TFP-UTRCH-ABD, red) localizes to the leading process during glial-guided neuronal migration. Stable F-actin increased in the proximal portion of the leading process when the Golgi apparatus (GalNAcT2-YFP, green) is translocated forward into this region. Images were acquired every 3 minutes. Total elapsed time is 1 hour and 19 minutes. Displayed in Figure 1B. Time is displayed as hours:minutes:seconds. Scale bar, 10 μm. (MOV 7 MB)

Additional file 3: Movie 3: Dynamic F-actin (TFP-Lifeact, red) localizes to the leading process during glial-guided neuronal migration. Dynamic F-actin increased in the proximal portion of the leading process when the Golgi apparatus (GalNAcT2-YFP, green) translocated forward into this region. Images were acquired every 3 minutes. Total elapsed time is 2 hours and 6 minutes. Displayed in Figure 1C. Time is displayed as hours:minutes:seconds. Scale bar, 10 μm. (MOV 4 MB)

Additional file 4: Movie 4: Two-dimensional intensity plot of the imaging sequence shown in Figure 1B. The neuron in this sequence was segmented with the custom-designed algorithm described in Materials and Methods. A line approximating the vector of migration is shown in grey and selected orthogonal planes of the segmentation are shown in black. The fluorescence intensity of TFP-UTRCH (red) and GalNAcT2-YFP (green) along the extent of the entire neuron are displayed as a curve above the approximate region of the segmented cell. The Golgi apparatus peak near the moves within the domain of leading process F-actin that flows down the leading process prior to cell soma movement. (ZIP 262 KB)

Additional file 5: Movie 5: Two-dimensional intensity plot of the imaging sequence shown in Figure 1C. The neuron in this sequence was segmented with the custom-designed algorithm described in Materials and Methods. A line approximating the vector of migration is shown in grey and selected orthogonal planes of the segmentation are shown in black. The fluorescence intensity of TFP-Lifeact (red) and GalNAcT2-YFP (green) along the extent of the entire neuron are displayed as a curve above the approximate region of the segmented cell. The Golgi apparatus peak near the moves within the domain of leading process F-actin that flows down the leading process prior to cell soma movement. (ZIP 246 KB)

Additional file 6: Movie 6: Blebbistatin (100 μM) inhibits Golgi (GalNAcT2-YFP, green) and somal translocation (labeled with H2B-mCherry, red) in a migrating neuron. Displayed in Figure 2A. Time is displayed as hours:minutes:seconds. Scale bar, 10 μm. (MOV 1 MB)

Additional file 7: Movie 7: Jasplakinolide (5 μM) inhibits Golgi (GalNAcT2-YFP, green) and somal translocation (labeled with H2B-mCherry, red) in a migrating neuron. Displayed in Figure 2A. Time is displayed as hours:minutes:seconds. Scale bar, 10 μm. (MOV 2 MB)

Additional file 8: Movie 8: Basal Golgi apparatus (GalNAcT2-YFP, green) movement is constant in control stationary neurons. CGNs were nucleofected with vectors encoding GalNAcT2-YFP (green) and H2B-mCherry (red) and time-lapse imaging was used to examine Golgi position every 10 minutes over the course of a 1-hour imaging experiment. The plane on the left was acquired a t = 0 and on the right t = 60. Displayed in Figure 3A. Time is displayed as hours:minutes:seconds. Scale bar, 10 μm. (MOV 1 MB)

Additional file 9: Movie 9: Basal Golgi apparatus (GalNAcT2-YFP, green) movement is reduced by myosin ii inhibition in stationary neurons. CGNs were nucleofected with vectors encoding GalNAcT2-YFP (green) and H2B-mCherry (red) and time-lapse imaging was used to examine Golgi position every 10 minutes over the course of a 1-hour imaging experiment where 100 mM blebbistatin was added to the bath. The Golgi was motile in the pre-treatment plane on the left and movement was reduced in CGNs treated with blebbistatin for 60 minutes in the plane on the right. Displayed in Figure 3B. Time is displayed as hours:minutes:seconds. Scale bar, 10 μm. (MOV 1 MB)

Additional file 10: Movie 10: Basal Golgi apparatus (GalNAcT2-YFP, green) movement is reduced by jasplakinolide treatment in stationary neurons. CGNs were nucleofected with vectors encoding GalNAcT2-YFP (green) and H2B-mCherry (red) and time-lapse imaging was used to examine Golgi position every 10 minutes over the course of a 1-hour imaging experiment where 5 μM jasplakinolide was added to the bath. The Golgi was motile in the pre-treatment plane on the left and movement was reduced in CGNs treated with jasplakinolide for 60 minutes in the plane on the right. Displayed in Figure 3C. Time is displayed as hours:minutes:seconds. Scale bar, 10 μm. (MOV 2 MB)

Additional file 11: Movie 11: The primary cilium, labeled with Arl13b-Venus (green) enters a dilated region near the proximal portion of the leading process before nuclear translocation (nucleus labeled with H2B-mCherry, red). Images were acquired every 2 minutes and 30 seconds. Total elapsed time is 57 minutes. Displayed in Figure 4A. Time is displayed as hours:minutes:seconds. Scale bar, 10 μm. (MOV 8 MB)

Additional file 12: Movie 12: The primary cilium, labeled with Arl13b-Venus (green) enters the proximal portion of the leading process in tandem with the mother centriole (labeled with PACT-KO2, red). Images were acquired every 3 minutes. Total elapsed time is 36 minutes. Displayed in Figure 4B. Time is displayed as hours:minutes:seconds. Scale bar, 10 μm. (MOV 2 MB)

Additional file 13: Movie 13: The primary cilium (labeled with Arl13b-KO2, red) is transiently embedded within the stable F-actin contractile domain (labeled by EGFP-UTRCH-ABD, green) at distinct stages of the migration cycle. Images were acquired every 5 minutes. Total elapsed time is 35 minutes. Displayed in Figure 5A. Time is displayed as hours:minutes:seconds. Scale bar, 10 μm. (MOV 2 MB)

Additional file 14: Movie 14: The primary cilium (labeled with Arl13b-KO2, red) is transiently embedded within the dynamic F-actin contractile domain (labeled by EGFP-Lifeact, green) at distinct stages of the migration cycle. Images were acquired every 3 minutes. Total elapsed time is 18 minutes. Displayed in Figure 5B. Time is displayed as hours:minutes:seconds. Scale bar, 10 μm. (MOV 1 MB)

Additional file 15: Movie 15: The primary cilium (labeled with Arl13b-KO2, red) co-localizes with myosin ii heavy chain (labeled by EGFP-Lifeact, green) during the first stroke of the CGN migration cycle. Images were acquired every 2 minutes. Total elapsed time is 12 minutes. Displayed in Figure 5C. Time is displayed as hours:minutes:seconds. Scale bar, 10 μm. (MOV 289 KB)

Additional file 16: Movie 16: Blebbistatin (100 μM) inhibits primary cilium (Arl13b-Venus, green) and somal translocation (labeled with H2B-mCherry, red) in a migrating neuron. Displayed in Figure 6A. Time is displayed as hours:minutes:seconds. Scale bar, 10 μm. (MOV 84 KB)

Additional file 17: Movie 17: Jasplakinolide (5 μM) inhibits primary cilium (Arl13b-Venus, green) and somal translocation (labeled with H2B-mCherry, red) in a migrating neuron. Displayed in Figure 6A. Time is displayed as hours:minutes:seconds. Scale bar, 10 μm. (MOV 1 MB)

Additional file 18: Figure S1: Myosin ii and F-actin dynamics and motor activity are required for coordinated movement of the Golgi and primary cilia. CGNs were transfected to express the Golgi label GalNAcT2-Venus (green), the primary cilia label Arl13b-mKO1 (red), and TFP-Lifeact (not shown). After 24 minutes of migration, 100 μM blebbistatin or 5 μM jasplakinolide were added to the culture, and imaging continued for a further 36 minutes. The outline of the cell at each time point is traced with a white dashed line. The soma and leading process (lp) are labeled in first frame of each sequence. The time-lapse panels show both drugs potently inhibited forward movement of the Golgi, primary cilia, and cell body. (TIFF 2 MB)

Additional file 19: Figure S2: Acute inhibition of myosin ii motor activity and F-actin dynamics have little effect on microtubule dynamics. CGNs were transfected to express the microtubule label Map2c-Venus. After 24 minutes of migration, 100 μM blebbistatin or 5 μM jasplakinolide were added to the culture, and imaging continued for a further 36 minutes. The soma and leading process (lp) are labeled in first frame of each sequence. The time-lapse panels and adaptive kymographs show both drugs do not grossly perturb the overall structure of the microtubule cytoskeleton. (TIFF 3 MB)

Additional file 20: Figure S3: Acute inhibition of myosin ii motor activity and F-actin dynamics have little effect on microtubule stability. CGNs were cultured for 24 hours *in vitro* and 100 μM blebbistatin or 5 μM jasplakinolide were added to the medium and cultured for a further 30 minutes (e.g., conditions similar to our live imaging experiments). The cultures were then fixed and singly stained for antibodies recognizing acetylated α-tubulin or tyrosinated α-tubulin (stable microtubules) and co-stained with antibodies recognizing β-tubulin and de-tyrosinated α-tubulin. There was no difference in staining for acetylated α-tubulin, tyrosinated α-tubulin (stable microtubules), or de-tyrosinated α-tubulin between control, blebbistatin-, or jasplakinolide-treated cultures, indicating that differences in organelle positioning elicited by cytoskeletal drug treatment are not associated with alterations in the balance of microtubule post-translational modifications. (TIFF 7 MB)

Additional file 21: Movie 18: Basal primary cilium (Arl13b-Venus, green) movement is constant in control stationary neurons. CGNs were nucleofected with vectors encoding Arl13b-Venus (green) and H2B-mCherry (red) and time-lapse imaging was used to examine primary cilium position every 10 minutes over the course of a 1-hour imaging experiment. The plane on the left was acquired a t = 0 and on the right t = 60. Displayed in Figure 7A. Time is displayed as hours:minutes:seconds. Scale bar, 10 μm. (MOV 2 MB)

Additional file 22: Movie 19: Basal primary cilium (Arl13b-Venus, green) movement is reduced by myosin ii inhibition in stationary neurons. CGNs were nucleofected with vectors encoding Arl13b-Venus (green) and H2B-mCherry (red), and time-lapse imaging was used to examine primary cilium position every 10 minutes over the course of a 1-hour imaging experiment where 100 mM blebbistatin was added to the bath. The primary cilium was motile in the pre-treatment plane on the left and movement was reduced in CGNs treated with blebbistatin for 60 minutes in the plane on the right. Displayed in Figure 7B. Time is displayed as hours:minutes:seconds. Scale bar, 10 μm. (MOV 1 MB)

Additional file 23: Movie 20: Basal primary cilium (Arl13b-Venus, green) movement is reduced by jasplakinolide treatment in stationary neurons. CGNs were nucleofected with vectors encoding Arl13b-Venus (green) and H2B-mCherry (red), and time-lapse imaging was used to examine primary cilium position every 10 minutes over the course of a 1-hour imaging experiment where 5 μM jasplakinolide was added to the bath. The primary cilium was motile in the pre-treatment plane on the left and movement was reduced in CGNs treated with jasplakinolide for 60 minutes in the plane on the right. Displayed in Figure 7C. Time is displayed as hours:minutes:seconds. Scale bar, 10 μm. (MOV 86 KB)

Additional file 24: Movie 21: JAM-C adhesions (labeled by JAM-C-pHluorin, green) localize to stable F-actin-rich (RFP-UTRCH-ABD, red) domains within the leading process and soma in migrating CGNs. Images were acquired every 5 minutes. Total elapsed time is 1 hour and 5 minutes. Displayed in Figure 8A. Time is displayed as hours:minutes:seconds. Scale bar, 10 μm. (MOV 2 MB)

Additional file 25: Movie 22: Two-dimensional intensity plot of the imaging sequence shown in Figure 8A. The neuron in this sequence was segmented with the custom-designed algorithm described in Materials and Methods. A line approximating the vector of migration is shown in grey and selected orthogonal planes of the segmentation are shown in black. The fluorescence intensity of RFP-UTRCH (red) and JAM-C-pHluorin (green) along the extent of the entire neuron are displayed as a curve above the approximate region of the segmented cell. The fluorescence intensities of f-actin and JAM-C adhesions are highly coincident in the leading process and cell body, indicating that JAM-C adhesions are localized to f-actin rich regions of migrating neurons. (MOV 14 MB)

Additional file 26: Movie 23: JAM-C adhesions (labeled by JAM-C-pHluorin, green) localize to dynamic F-actin-rich (RFP-Lifeact, red) domains within the leading process and soma in migrating CGNs. Images were acquired every 2 minutes. Total elapsed time is 18 minutes. Displayed in Figure 8B. Time is displayed as hours:minutes:seconds. Scale bar, 10 μm. (ZIP 1 MB)

Additional file 27: Movie 24: Two-dimensional intensity plot of the imaging sequence shown in Figure 8B. The neuron in this sequence was segmented with the custom-designed algorithm described in Materials and Methods. A line approximating the vector of migration is shown in grey and selected orthogonal planes of the segmentation are shown in black. The fluorescence intensity of RFP-Lifeact (red) and JAM-C-pHluorin (green) along the extent of the entire neuron are displayed as a curve above the approximate region of the segmented cell. The fluorescence intensities of f-actin and JAM-C adhesions are highly coincident in the leading process and cell body, indicating that JAM-C adhesions are localized to f-actin-rich regions of migrating neurons. (MOV 15 MB)

Additional file 28: Movie 25: Cadm3 adhesions (labeled by Cadm3-pHluorin, green) localize to stable F-actin-rich (RFP-UTRCH-ABD, red) domains within the leading process and soma in migrating CGNs. Images were acquired every 3 minutes. Total elapsed time is 42 minutes. Displayed in Figure 8C. Time is displayed as hours:minutes:seconds. Scale bar, 10 μm. (MOV 448 KB)

Additional file 29: Movie 26: Two-dimensional intensity plot of the imaging sequence shown in Figure 8C. The neuron in this sequence was segmented with the custom-designed algorithm described in Materials and Methods. A line approximating the vector of migration is shown in grey and selected orthogonal planes of the segmentation are shown in black. The fluorescence intensity of RFP-UTRCH (red) and Cadm3-pHluorin (green) along the extent of the entire neuron are displayed as a curve above the approximate region of the segmented cell. The fluorescence intensities of f-actin and Cadm3 adhesions are highly coincident in the leading process and cell body, indicating that Cadm3 adhesions are localized to f-actin rich regions of migrating neurons. (MOV 12 MB)

Additional file 30: Movie 27: Cadm3 adhesions (labeled by Cadm3-pHluorin, green) localize to dynamic F-actin (RFP-Lifeact, red) rich domains within the leading process and soma in migrating CGNs. Images were acquired every 3 minutes. Total elapsed time is 1 hour and 45 minutes. Displayed in Figure 8D. Time is displayed as hours:minutes:seconds. Scale bar, 10 μm. (MOV 1 MB)

Additional file 31: Movie 28: Two-dimensional intensity plot of the imaging sequence shown in Figure 8D. The neuron in this sequence was segmented with the custom-designed algorithm described in Materials and Methods. A line approximating the vector of migration is shown in grey and selected orthogonal planes of the segmentation are shown in black. The fluorescence intensity of RFP-Lifeact (red) and Cadm3-pHluorin (green) along the extent of the entire neuron are displayed as a curve above the approximate region of the segmented cell. The fluorescence intensities of f-actin Cadm3 adhesions are highly coincident in the leading process and cell body, indicating that Cadm3 adhesions are localized to f-actin-rich regions of migrating neurons. (MOV 9 MB)

Additional file 32: Movie 29: Blebbistatin (100 μM) slows JAM-C (labeled with JAM-C pHluorin) adhesion dynamics and somal translocation in a migrating neuron. Displayed in Figure 9A. Time is displayed as hours:minutes:seconds. Scale bar, 10 μm. (MOV 1 MB)

Additional file 33: Movie 30: Jasplakinolide (5 μM) slows JAM-C (labeled with JAM-C pHluorin) adhesion dynamics and somal translocation in a migrating neuron. Displayed in Figure 9B. Time is displayed as hours:minutes:seconds. Scale bar, 10 μm. (MOV 973 KB)

Additional file 34: Movie 31: Blebbistatin (100 μM) slows Cadm3 (labeled with Cadm3-pHluorin) adhesion dynamics and somal translocation in a migrating neuron. Displayed in Figure 9C. Time is displayed as hours:minutes:seconds. Scale bar, 10 μm. (MOV 501 KB)

Additional file 35: Movie 32: Jasplakinolide (5 μM) slows Cadm3 (labeled with Cadm3-pHluorin) adhesion dynamics and somal translocation in a migrating neuron. Displayed in Figure 9D. Time is displayed as hours:minutes:seconds. Scale bar, 10 μm. (MOV 1 MB)

Additional file 36: Figure S4: Model of actomyosin action on the Golgi, primary cilia, and adhesion molecules during CGN migration. **(A)** During glial-guided of migration of CGNs are polarized in the direction of migration (arrow). A leading process extends in the direction of migration, cytoplasmic organelles like the Golgi are often found where the leading process tapers into the neuronal cell body and a gradient of adhesive contacts exist where neurons contact a migration substrate (glial fiber is shaded grey, adhesions are depicted in red) **(B)** As the Golgi is not docked at the plasma membrane near the actomyosin cortex, perhaps actomyosin acts to position Golgi-attached microtubules to elicit Golgi positioning events. **(C)** As primary cilia and cell adhesion molecules are located at the plasma membrane near the actomyosin-rich cortex, positioning of these cellular elements likely represents a local activity of the actomyosin cytoskeleton. (TIFF 2 MB)

## Abbreviations

CGN: Cerebellar granule neuron
FRAP: Fluorescence recovery after photobleaching
KO2: Kusibira orange
MGE: Medial ganglionic eminence
RFP: Red fluorescent protein
TFP: Teal fluorescent protein
UTRCH-ABD: Utrophin actin-binding domain
YFP: Yellow fluorescent protein.
