# Supplementary figures and images for: Leading-process actomyosin coordinates organelle positioning and adhesion receptor dynamics in radially migrating cerebellar granule neurons
Source: Neural Dev. 2014 Dec 2;9:26. doi: 10.1186/1749-8104-9-26 (PMC4289176; doi:10.1186/1749-8104-9-26)

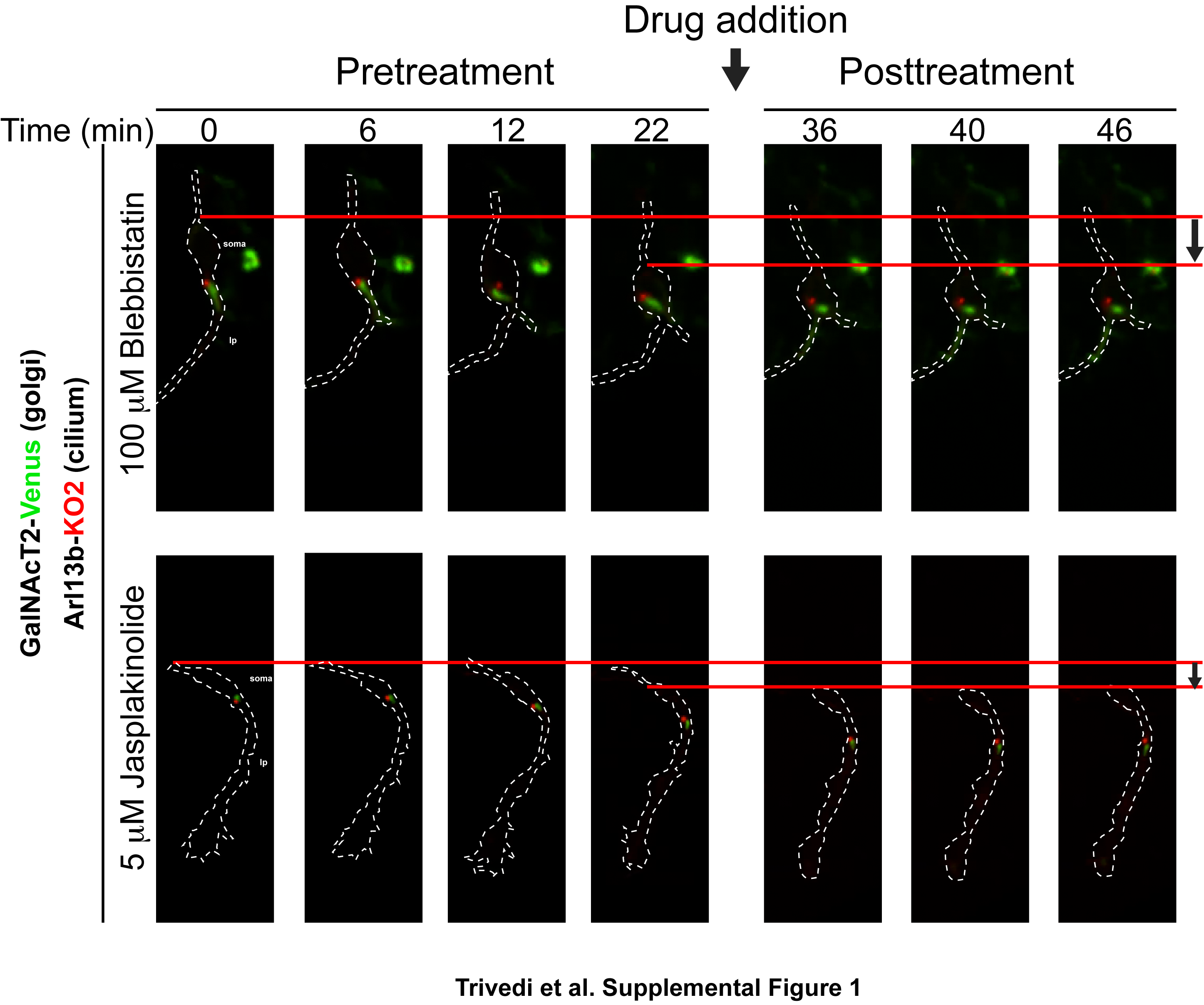

Supplement: Supplementary file 18 — Additional file 18: Figure S1: Myosin ii and F-actin dynamics and motor activity are required for coordinated movement of the Golgi and primary cilia. CGNs were transfected to express the Golgi label GalNAcT2-Venus (green), the primary cilia label Arl13b-mKO1 (red), and TFP-Lifeact (not shown). After 24 minutes of migration, 100 μM blebbistatin or 5 μM jasplakinolide were added to the culture, and imaging continued for a further 36 minutes. The outline of the cell at each time point is traced with a white dashed line. The soma and leading process (lp) are labeled in first frame of each sequence. The time-lapse panels show both drugs potently inhibited forward movement of the Golgi, primary cilia, and cell body. (TIFF 2 MB) [file 13064_2014_269_MOESM18_ESM.tiff]

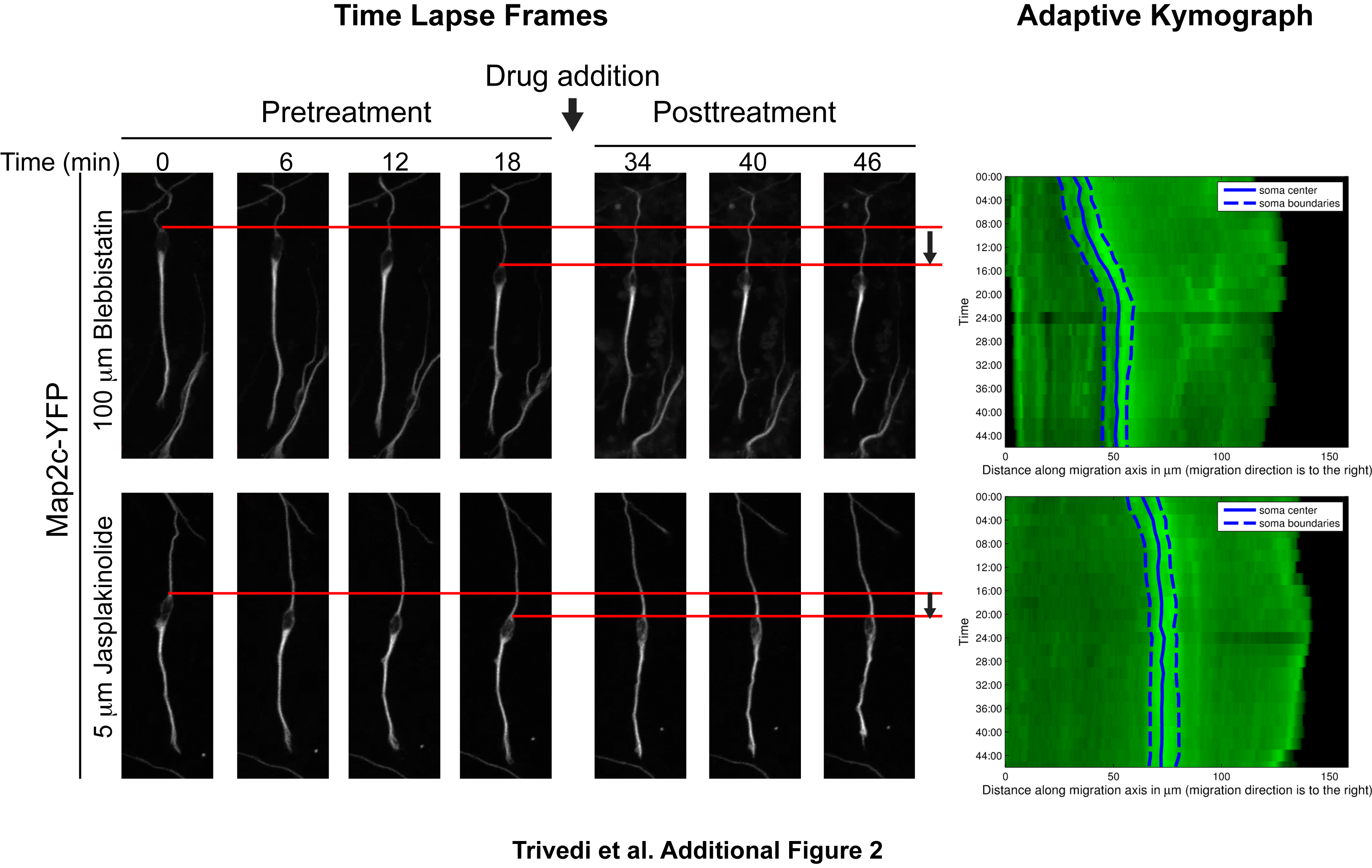

Supplement: Supplementary file 19 — Additional file 19: Figure S2: Acute inhibition of myosin ii motor activity and F-actin dynamics have little effect on microtubule dynamics. CGNs were transfected to express the microtubule label Map2c-Venus. After 24 minutes of migration, 100 μM blebbistatin or 5 μM jasplakinolide were added to the culture, and imaging continued for a further 36 minutes. The soma and leading process (lp) are labeled in first frame of each sequence. The time-lapse panels and adaptive kymographs show both drugs do not grossly perturb the overall structure of the microtubule cytoskeleton. (TIFF 3 MB) [file 13064_2014_269_MOESM19_ESM.tiff]

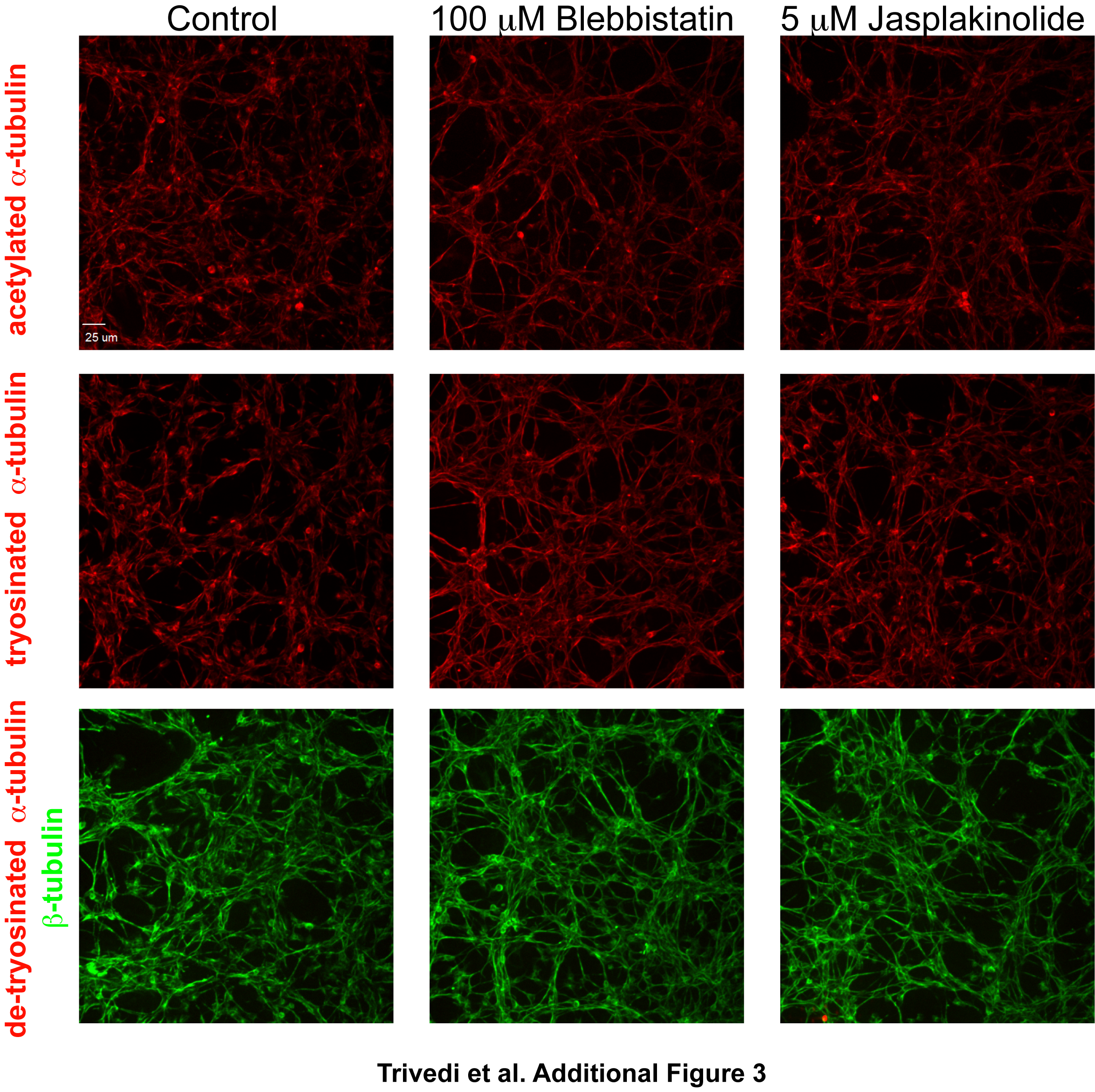

Supplement: Supplementary file 20 — Additional file 20: Figure S3: Acute inhibition of myosin ii motor activity and F-actin dynamics have little effect on microtubule stability. CGNs were cultured for 24 hours in vitro and 100 μM blebbistatin or 5 μM jasplakinolide were added to the medium and cultured for a further 30 minutes (e.g., conditions similar to our live imaging experiments). The cultures were then fixed and singly stained for antibodies recognizing acetylated α-tubulin or tyrosinated α-tubulin (stable microtubules) and co-stained with antibodies recognizing β-tubulin and de-tyrosinated α-tubulin. There was no difference in staining for acetylated α-tubulin, tyrosinated α-tubulin (stable microtubules), or de-tyrosinated α-tubulin between control, blebbistatin-, or jasplakinolide-treated cultures, indicating that differences in organelle positioning elicited by cytoskeletal drug treatment are not associated with alterations in the balance of microtubule post-translational modifications. (TIFF 7 MB) [file 13064_2014_269_MOESM20_ESM.tiff]

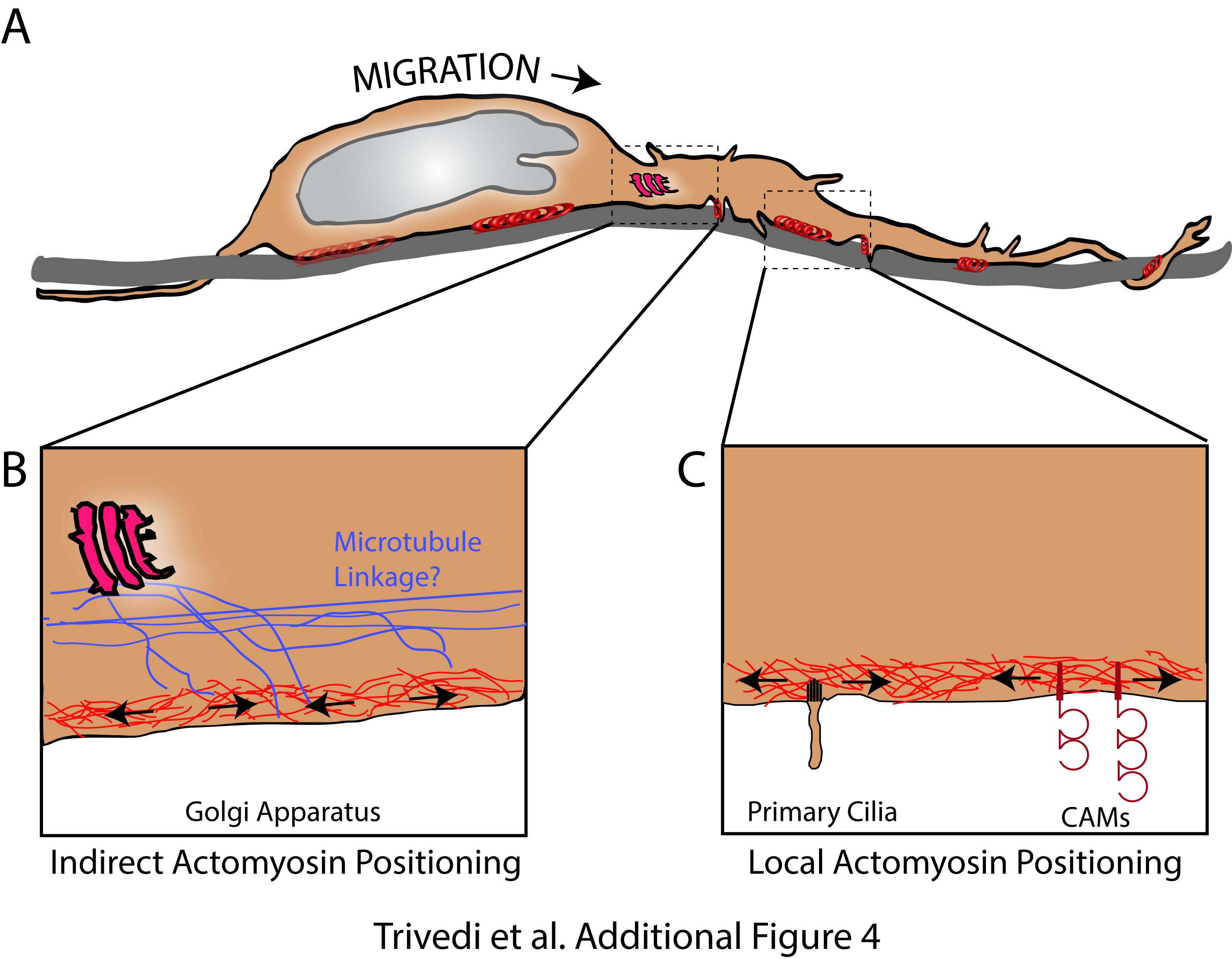

Supplement: Supplementary file 36 — Additional file 36: Figure S4: Model of actomyosin action on the Golgi, primary cilia, and adhesion molecules during CGN migration. (A) During glial-guided of migration of CGNs are polarized in the direction of migration (arrow). A leading process extends in the direction of migration, cytoplasmic organelles like the Golgi are often found where the leading process tapers into the neuronal cell body and a gradient of adhesive contacts exist where neurons contact a migration substrate (glial fiber is shaded grey, adhesions are depicted in red) (B) As the Golgi is not docked at the plasma membrane near the actomyosin cortex, perhaps actomyosin acts to position Golgi-attached microtubules to elicit Golgi positioning events. (C) As primary cilia and cell adhesion molecules are located at the plasma membrane near the actomyosin-rich cortex, positioning of these cellular elements likely represents a local activity of the actomyosin cytoskeleton. (TIFF 2 MB) [file 13064_2014_269_MOESM36_ESM.tiff]
